# Comparing the Acceptability and Quality of Intervention Modalities for Suicidality in the Emergency Department: Randomized Feasibility Trial

**DOI:** 10.2196/49783

**Published:** 2023-10-24

**Authors:** Celine Larkin, Bengisu Tulu, Soussan Djamasbi, Roscoe Garner, Fatima Varzgani, Mariam Siddique, John Pietro, Edwin D Boudreaux

**Affiliations:** 1 Department of Emergency Medicine University of Massachusetts Chan Medical School Worcester, MA United States; 2 The Business School Worcester Polytechnic Institute Worcester, MA United States

**Keywords:** suicide, self-harm, emergency department, mobile app, intervention, mobile technology, safety planning, safety, suicidal, emergency, mHealth, mobile health, mental health, mobile phone

## Abstract

**Background:**

Emergency departments (EDs) manage many patients with suicide risk, but effective interventions for suicidality are challenging to implement in this setting. ReachCare is a technology-facilitated version of an evidence-based intervention for suicidal ED patients. Here, we present findings on the acceptability and quality of ReachCare in the ED, as well as a comparison of these measures across 3 potential delivery modalities

**Objective:**

Our aim was to test the feasibility of the ReachCare intervention in its entirety through conducting a pilot study with patients presenting with suicidality to the ED. We tested three different ways of receiving the ED-based components of ReachCare: (1) self-administered on the tablet app using a chatbot interface, (2) administered by an in-person clinician, or (3) administered by a telehealth clinician.

**Methods:**

In total, 47 ED patients who screened positive for suicide risk were randomly allocated to receive one of three delivery modalities of ReachCare in the ED: (1) self-administered on the patient-facing tablet app with a chatbot interface, (2) delivered by an in-person clinician, or (3) delivered by a telehealth clinician, with the latter two using a clinician-facing web app. We measured demographic and clinical characteristics, acceptability and appropriateness of the intervention, and quality and completeness of the resulting safety plans.

**Results:**

Patients assigned high ratings for the acceptability (median 4.00/5, IQR 4.00-4.50) and appropriateness (median 4.00/5, IQR 4.00-4.25) of ReachCare’s ED components, and there were no substantial differences across the 3 delivery modalities [H(acceptability)=3.90, *P*=.14; H(appropriateness)=1.05, *P*=.59]. The self-administered modality took significantly less time than the 2 clinician modalities (H=27.91, *P*<.001), and the usability of the self-administered version was in the “very high” range (median 93.75/100, IQR 80.00-97.50). The safety plans created across all 3 modalities were high-quality (H=0.60, *P*=.74).

**Conclusions:**

Patients rated ReachCare in the ED as highly acceptable and appropriate regardless of modality. Self-administration may be a feasible way to ensure patients with suicide risk receive an intervention in resource constrained EDs. Limitations include small sample size and demographic differences between those enrolled versus not enrolled. Further research will examine the clinical outcomes of patients receiving both the in-ED and post-ED components of ReachCare.

**Trial Registration:**

ClinicalTrials.gov NCT04720911; https://clinicaltrials.gov/ct2/show/NCT04720911

## Introduction

After a brief decline in 2019-2020 [1,2], suicide rates have again begun to rise in the United States [1]. Each year, there are over 1 million suicide-related visits to emergency departments (EDs) in the United States [3], and EDs represent a feasible venue to screen and assess suicide risk [4,5]. However, once risk is detected, many ED patients with suicide risk receive suboptimal care [6].

Several effective interventions have been developed for this patient population [7]. The Emergency Department Safety Assessment and Follow-up Evaluation (ED-SAFE) intervention [8], for example, featured paper-based self-administered safety planning and resource lists in the ED; this was followed by intensive telephone outreach to patients and a chosen significant other in the 12 months after discharge. The telephone counseling involved safety planning, outpatient treatment engagement, values clarification and life planning, and family communication and coordination. The ED-SAFE intervention was associated with a 30% reduction in the total number of suicide attempts in the intervention group compared to treatment-as-usual [8]. Despite its promise and cost-effectiveness [9], the ED-SAFE intervention was resource-intensive and difficult to sustain without research support. Such interventions routinely struggle to “cross the chasm” to become routine care [10].

Clinicians and leadership recognize that ED-based care for suicidality needs to improve but often face substantial constraints in effecting change [11,12]. The dearth of effective care for suicidality in the ED can be attributed to a wide range of factors, including a hectic milieu focused on patient flow [13], a shortage of behavioral health (BH) providers [14], and a shortfall in suicide-related skills and confidence among ED clinicians [15,16]. With these barriers in mind, we set out to adapt the ED-SAFE intervention to become “ReachCare,” leveraging technology to make the intervention scalable in resource-constrained EDs, while avoiding a corresponding loss in fidelity.

We previously outlined the user-centered development and testing of the patient-facing technology aspects of the ReachCare intervention [17]. These technologies include a tablet-based app for use within the ED (that allows a patient to build a high-quality safety plan in the absence of a BH clinician) and a patient- and family-facing mobile app (that houses the patient’s safety plan and life plan, helpline resources, psychoeducation, distractions, and a BH provider search engine). In that study, user-testing with current ED patients presenting with suicidality demonstrated high usability and acceptability of the technologies [17]. ReachCare is further supported by interoperable clinician-facing software that provides tips, call structure, and note-taking for clinicians, as well as allowing them to update and share patients’ therapeutic tools during follow-up phone calls. This clinician-facing technology was designed to be used in-person or via telehealth.

We conducted a feasibility trial with patients presenting with suicidality to the ED to develop and test implementation strategies for the ReachCare intervention that will be used in a future effectiveness trial. We tested three different modalities of delivering the ED-based components of ReachCare, randomizing patients to receive: (1) self-administered on the tablet app using a chatbot interface, (2) administered by an in-person clinician, or (3) administered by a telehealth clinician. Here, we present the findings from the ED-based portion of the feasibility trial, namely the acceptability and appropriateness of ReachCare in the ED overall, the quality and completeness of safety plans created in the ED, intervention duration, and a comparison of these measures across the 3 potential delivery modalities.

## Methods

### Ethical Considerations

This study was approved by the institutional review board of the University of Massachusetts Chan Medical School on May 19, 2020, under docket number H00020238.

### Study Design

This study was a feasibility trial of the ReachCare intervention, in which eligible patients were randomized to receive ReachCare in the ED in one of three ways: (1) self-administered on the tablet app using a chatbot interface, (2) administered by an in-person clinician, or (3) administered by a telehealth clinician. Randomization was on a 1:1:1 ratio based on blocks of 6: randomization tables were prepared by a team member who was not involved in data collection and was uploaded to the Research Electronic Data Capture (Vanderbilt University) data collection system. Allocation was done after the baseline research interview to preserve blinding of the research assistant collecting the baseline demographic and clinical measures. Further, because the research assistant had to know the allocation in order to facilitate the intervention, blinding was not possible for the acceptability measures, which were completed after the intervention. The quality rating of the safety plans was completed after completion of the trial by an assessor blinded to allocation (see more detail under the Measures section). The target sample size of 45 (15 participants per arm) was set based on previous literature recommending sample sizes between 24 and 50 for feasibility studies [18,19].

### Recruitment

This study took place in the ED of a large teaching hospital in the northeastern United States. Over a 6-month period, we sought to enroll patients who presented to the ED and screened positive for suicidality on the Patient Safety Screener (PSS) [20]. The PSS is used universally and routinely at the enrollment ED: a positive screen is defined as active ideation in the past 2 weeks or a suicide attempt in the past 6 months. Patients were considered for inclusion if they (1) screened positive for suicidality on the PSS, (2) were aged 18 years or older, (3) were cognitively and medically able to consent and participate, (4) owned a smartphone, and (5) had a stable mailing address to receive compensation. Patients were excluded if they were (1) a prisoner or in police custody, (2) overly agitated or violent, or (3) on precautions for infectious disease, such as COVID-19.

Potentially eligible patients were approached by a research assistant in the ED’s medical or psychiatric area and were screened to confirm eligibility. The research assistant then explained the study: patients were informed as to what the study entailed, procedures to protect confidentiality and privacy, their right to withdraw at any time, and the potential risks and benefits of the study. If the patient was still interested in participating, the research assistant then administered a consent mini-quiz to test the patient’s understanding of the information and invited them to read and sign an informed consent form. Participants then completed baseline research measures, were randomly allocated to 1 of 3 arms, participated in the ED portion of the intervention, and then completed acceptability measures. Participants received a US $30 gift-card for completion of the baseline research interview.

### Intervention

**Figure 1:**
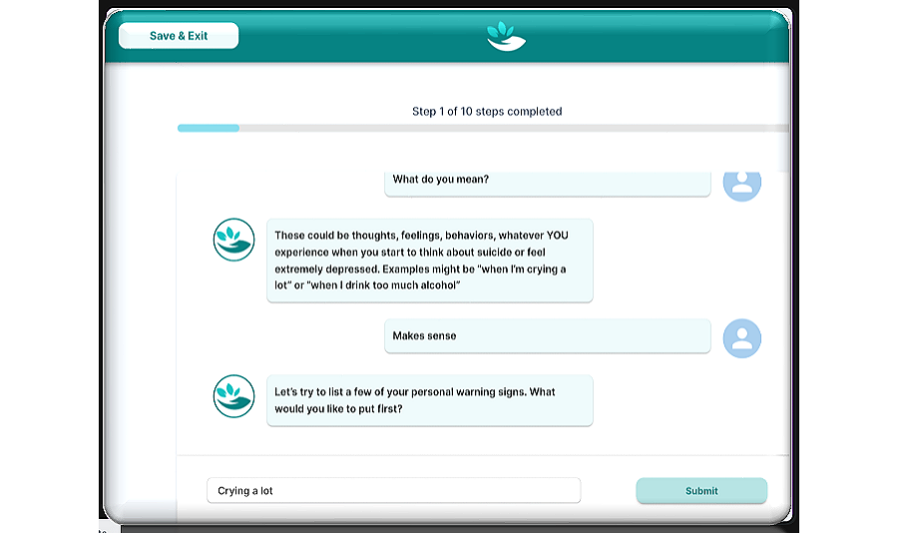
Screenshot of the ReachCare tablet-based app's safety planning functionality.

**Figure 2:**
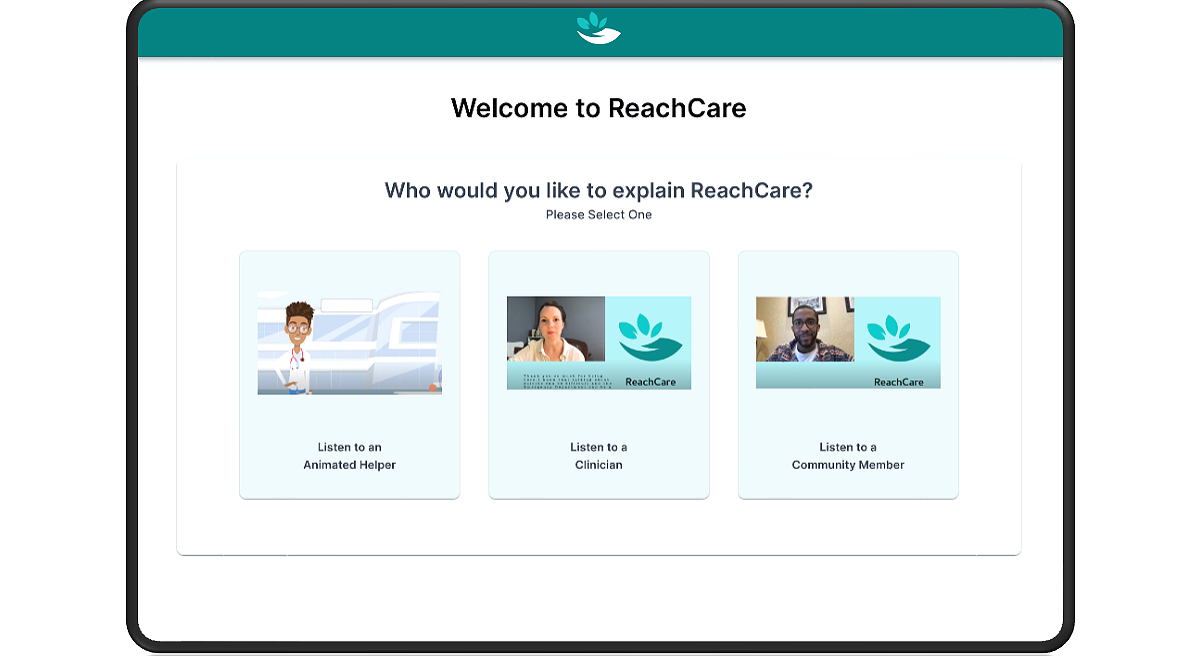
Screenshot of the ReachCare tablet-based app’s video functionality.

The ED-based portion of the ReachCare intervention focuses on creating a 6-step safety plan [21]. The safety plan involves working with the patient to identify warning signs of an impending suicidal crisis, internal coping strategies, social supports and places for distraction, social supports for help, professionals for help, and making the environment safer by limiting access to lethal means (Figure 1). In the self-administered arm of the trial, patients engaged with a tablet-based app: they watched an introductory video (with the choice of watching a clinician, community member, or animated character deliver the same information; Figure 2) and then proceeded to create a safety plan by interacting with a chatbot-style interface that presented cues and questions, to which the patient typed in personalized answers [17]. Once the safety plan had been created, the patient had the opportunity to review and edit it, before watching an outro video explaining the next steps in their care. In contrast, in the clinician in-person and telehealth arms, the patient completed safety planning with the clinician. In those arms, the clinician used the ReachCare clinician portal, which provides clinician-facing cues, suggested phrasing, and structure to introduce the intervention to the patient and to create a safety plan with them.

### Measures

Our measures included a variety of modalities, including patient self-report via interview, medical chart review, researcher measurement (of duration of intervention), and researcher rating (of safety plan quality).

We assessed several demographic variables by patient self-report, namely gender, age, race, ethnicity, psychiatric diagnoses, BH treatments received in the past 3 months, access to lethal means, and smartphone usage. We assessed mobile technology self-efficacy by patient self-report using 3 items from Balapour et al [22]. The 15-item version of the Interpersonal Needs Questionnaire [23] was used to assess patient’s self-reported thwarted belonginess and perceived burdensomeness, key drivers of suicidality. The Interpersonal Needs Questionnaire includes a 7-point Likert Scale and has good psychometric properties [24]. We used the Columbia Suicide Severity Rating Scale to classify patient’s self-reported recent and past suicidal ideation and suicidal behavior [25] and suicide-related impulsivity was assessed using 2 items from Mccullumsmith et al [26].

We used the medical record to extract the patient’s insurance status and the circumstances of their current presentation (chief complaint, involuntary hold status, and suicide screening result). We also extracted information regarding the care they received in the ED beyond the research intervention, that is, whether they had received each of the following: a BH evaluation; referral to outpatient BH; a structured suicide risk assessment; constant observation; or hospital admission.

We investigated acceptability and appropriateness, usability (for the self-administered arm only), duration of intervention in minutes, and quality of safety plans. Acceptability and appropriateness were measured using Weiner et al’s [27] 4-item Acceptability of Intervention measure and Intervention Appropriateness Measure respectively. Both instruments are on a 4-point Likert-type scale and have good reliability and validity [27]. Usability was assessed using the System Usability Scale [28]: this scale has 10 items, 5 of which are reverse-scored, and is the standard in usability research. The range of possible scores on the System Usability Scale is from 0 to 100. The duration of the intervention was timed by the research assistant and rounded to the closest minute. The quality of the safety plans was rated using the Safety Planning Intervention Scoring Algorithm-Brief [29], which assesses whether the safety plan has content in each line and whether that content is sufficiently specific. Each line receives a rating of 0 (no content), 1 (poor content), or 2 (satisfactory content), which is summed to create a total quality score. This quality rating was completed by a researcher who was blinded to the allocation of the patient. This scale’s psychometric properties have not been established but, in our sample, the value for Cronbach α for the 19-item scale was α=.70, indicating acceptable internal consistency.

### Analyses

For the first part of the results, we summarized patient and encounter characteristics using descriptive statistics. We then examined differences in implementation measures (median duration, acceptability, appropriateness, and quality) across the 3 arms: these variables were not normally distributed so we used independent samples Kruskal-Wallis tests including post hoc tests as appropriate. Finally, we summarize completeness of the safety plans in each arm using descriptive statistics. All tests were 2-sided with α set at *P*<.05. Analyses were completed in SPSS (version 28; IBM Corp) [30].

## Results

### Characteristics of Sample

**Figure 3:**
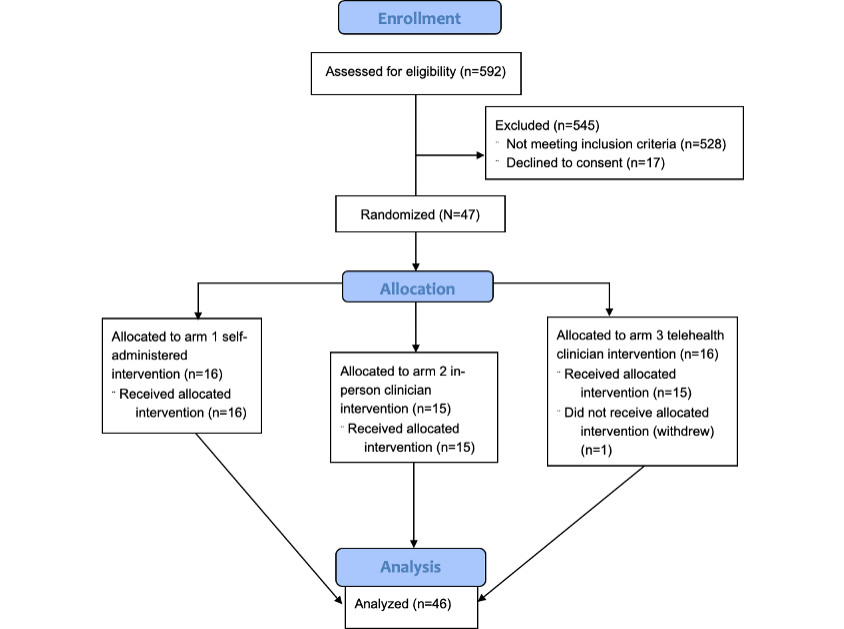
CONSORT (Consolidated Standards of Reporting Trials) flowchart showing progress of participants through the trial.

Figure 3 illustrates the patient enrollment flow. A total of 592 patients screened positive for suicide risk during research shifts and their medical charts were reviewed for initial eligibility: 144 of these were approached. Common reasons for nonapproach included the patient being: too ill, cognitively unable, agitated, without a stable mailing address, and being on infection control precautions. Of the 144 patients approached, 78 (55%) agreed to complete verbal eligibility screening. Of these, 64 were confirmed eligible, of whom 47 patients agreed to participate and were randomized: 16 participants to “arm 1 self-administered,” 15 participants to “arm 2 clinician in-person,” and 16 to “arm 3: telehealth clinician” (Figure 2). In total, 46 participants completed the intervention and all research measures; 1 patient allocated to arm 3 (telehealth clinician) withdrew during the intervention. Comparing those enrolled (N=47) to other patients who were screened for potential participation (n=545), those who were enrolled were more likely to be female (24/47, 51% vs 238/545, 44%), non-Hispanic (40/47, 85% vs 433/545, 79%), White (38/47, 81% vs 388/545, 71%), and younger (median 27.0, IQR 23-37 years vs 39.6, IQR 29-52 years). The characteristics of the enrolled sample are summarized in Tables 1 and 2 and are presented by study arm in Multimedia Appendix 1. Participants demonstrated high prevalence of depressive and anxiety disorders, high levels of smartphone use and self-efficacy, and high levels of perceived burdensomeness and thwarted belongingness.

Features of the participants’ index ED visit are summarized in Table 3, and are presented by study arm in Multimedia Appendix 2. Most participants were enrolled in the psychiatric area of the ED, almost all had a psychiatric chief complaint, and many were in the ED on an involuntary hold. Most participants received a BH evaluation during their ED visit and subsequent admission to a psychiatric facility.

**Table 1:** Patient characteristics at baseline (N=47).

Categorical variables		Patients, n (%)
**Gender**		
	Female	24 (51.1)
	Male	20 (42.6)
	Nonbinary	3 (6.4)
**Age (years)**		
	18-24	16 (34.0)
	25-34	17 (36.2)
	35+	14 (28.8)
**Race (check all that apply)**		
	White	38 (80.9)
	Other	5 (10.6)
	Black or African American	4 (8.5)
	Asian	2 (4.3)
	American Indian or Alaskan Native	1 (2.1)
	Native Hawaiian or Pacific Islander	0 (0)
**Ethnicity**		
	Non-Hispanic or Latinx	40 (85.1)
	Hispanic or Latinx	7 (14.9)
**Insurance**		
	Private	23 (48.9)
	Public (eg, Medicare and Medicaid)	19 (40.4)
	None or self-pay	5 (10.6)
**Psychiatric diagnoses (check all that apply)**		
	Depressive disorder	40 (85.1)
	Anxiety disorder	38 (80.9)
	Trauma or stress-related	24 (51.1)
	ADHD^a^	21 (44.7)
	Substance use disorder	16 (34.0)
	Personality disorder	12 (25.5)
	Eating disorder	5 (10.6)
	Schizophrenia or psychotic disorder	3 (6.4)
	Autism spectrum disorder	2 (4.3)
	Other	2 (4.3)
**Suicidal behavior**		
	Past-week active ideation	37 (78.7)
	Lifetime attempt	30 (63.8)
	Current attempt	7 (14.9)
**BH^b^ treatments received in past 3 months**		
	Prescription medication	37 (78.7)
	Individual therapy	27 (57.4)
	Partial hospitalization or intensive outpatient	17 (36.2)
	BH ED^c^ visit	12 (25.5)
	Group support	11 (23.4)
	Inpatient psychiatric hospitalization	9 (19.1)
	Helpline support	9 (19.1)
	Other	9 (19.1)
	Family therapy or couples counseling	4 (8.5)
**Lethal means**		
	Access to medication	40 (85.1)
	Access to firearm	0 (0)
**Smartphone use in past week per day**		
	>3 h a day	35 (74.5)
	30 min to 3 h	9 (19.1)
	<30 min a day	1 (2.1)
	Rarely or never	2 (4.3)

^a^ADHD: attention-deficit/hyperactivity disorder.

^b^BH: behavioral health.

^c^ED: emergency department.

**Table 2:** Patient characteristics at baseline (N=47).

Continuous variables		Mean (SD)
**Smartphone self-efficacy**		
	I can use smartphone technology if there was no one around to tell me what to do (max score 5)	4.62 (0.68)
	I can use smartphone technology even if I have never used a similar technology before (max score 5)	4.15 (1.02)
	I am confident that I can effectively open and use an app on my smartphone (max score 5)	4.79 (0.46)
**Hypothesized mechanisms of action**		
	INQ^a^ perceived burdensomeness subscale	19.57 (10.04)
	INQ thwarted belongingness subscale	33.83 (13.24)
	BAS^b^-drive subscale	11.17 (3.01)
	Suicide-related impulsivity	3.02 (1.35)

^a^INQ: Interpersonal Needs Questionnaire.

^b^BAS: behavioral activation scale.

**Table 3:** Characteristics of index ED^a^ visit (N=47).

			Patients, n (%)
**Enrollment location**			
	Psychiatric ED	30 (63.8)	
	Medical ED	17 (36.2)	
**Chief complaint**			
	Suicidality	35 (74.5)	
	Nonsuicide psychiatric	11 (23.4)	
	Nonpsychiatric	1 (2.2)	
**Involuntary BH^b^ hold**			
	Yes	41 (87.2)	
	No	6 (12.8)	
**Patient Safety Screener result (check all that apply)**			
	Positive for active ideation in past 2 wk	43 (91.5)	
	Positive for suicide attempt in past 6 mo	15 (31.9)	
**BH Evaluation**			
	Yes	40 (85.1)	
	No	7 (14.9)	
**Referral to outpatient BH**			
	Yes	1 (2.1)	
	No	46 (97.9)	
**Structured suicide assessment**			
	Yes	26 (55.3)	
	No	21 (44.7)	
**Under observation**			
	Yes	45 (95.7)	
	No	2 (4.3)	
**Disposition**			
	Psychiatric admission or transfer	29 (61.7)	
	Home	16 (34.0)	
	Other or not documented	2 (4.3)	

^a^ED: emergency department.

^b^BH: behavioral health.

### Implementation Outcomes of ReachCare in the ED

Table 4 summarizes the response of participants to ReachCare in the ED. Participants rated the acceptability and appropriateness of ReachCare in the ED highly, with no significant difference across the 3 delivery modalities. The usability of the self-administered (chatbot) modality was also rated very highly by participants, and post hoc tests showed that the self-administered arm took significantly less time to complete than the 2 clinician arms. The overall quality of the safety plans generated were not significantly different across the 3 arms (Table 4).

Table 5 summarizes the completeness of each step of the safety plans, compared across the 3 study arms. There were no statistical differences in the completeness of each step across arms, but Step 2 tended to be more complete in the self-administered arm (H=5.14, *P*=.08) and Step 5 tended to be more complete in the 2 clinician arms (H=4.96, *P*=.08).

**Table 4:** Implementation outcomes postintervention (n=46) by treatment arm.

			Median (IQR)		Kruskal-Wallis H			*P* value
**Acceptability of intervention (patient report, range 1-5)**						3.90		.14
	Overall	4.00 (4.00-4.50)						
	Arm 1 self	4.00 (3.81-4.56)						
	Arm 2 in-person	4.25 (4.00-4.50)						
	Arm 3 telehealth	4.00 (3.75-4.50)						
**Appropriateness of intervention (patient report, range 1-5)**						1.05		.59
	Overall	4.00 (4.00-4.25)						
	Arm 1 self	4.25 (3.75-4.25)						
	Arm 2 in-person	4.00 (4.00-4.50)						
	Arm 3 telehealth	4.00 (4.00-4.25)						
**System Usability Scale (patient report, range 0-100)**						N/A^a^		N/A
	Arm 1 only	93.75 (80.00-97.50)						
**Duration of intervention (researcher-measured, min)**						27.91		<.001^b^
	Overall	34.50 (16.50-41.75)						
	Arm 1 self	15.00 (14.75-17.75)						
	Arm 2 in-person	38.00 (33.50-41.75)						
	Arm 3 telehealth	42.50 (34.50-53.38)						
**Quality of safety plan (researcher-rated, range 0-38)**						0.60		.74
	Overall	30.00 (26.00-33.00)						
	Arm 1 self	30.00 (26.00-32.75)						
	Arm 2 in-person	30.00 (24.00-33.00)						
	Arm 3 telehealth	28.00 (26.00-34.00)						

^a^N/A: not applicable.

^b^Post hoc tests showed arm 1 had significantly shorter duration than arms 2 and 3 (*P*<.001).

**Table 5:** Safety plan completeness by step overall and by treatment arms (n=46).

Safety plan step	Number of lines with content, median (IQR)					H		*P* value
	Overall (all 3 arms)	Arm 1 self-administered	Arm 2 clinician in-person	Arm 3 clinician telehealth				
Step 1 warning signs (out of 3 lines)	3.0 (3.0-3.0)	3.0 (3.0-3.0)	3.0 (3.0-3.0)	3.0 (3.0-3.0)	1.28		.53	
Step 2 coping strategies (out of 3 lines)	3.0 (2.0-3.0)	3.0 (3.0-3.0)	3.0 (2.0-3.0)	2.0 (2.0-3.0)	5.14		.08	
Step 3 people or places for distraction (out of 4 lines)	3.0 (2.8-4.0)	4.0 (2.0-4.0)	3.0 (3.0-4.0)	3.0 (2.0-4.0)	1.02		.60	
Step 4 people for help (out of 3 lines)	2.0 (1.0-2.3)	2.0 (1.3-3.0)	2.0 (1.0-3.0)	2.0 (1.0-2.0)	1.12		.57	
Step 5 professionals for help (out of 4 lines)	3.0 (3.0-4.0)	3.0 (2.0-3.0)	3.0 (3.0-4.0)	3.0 (3.0-4.0)	4.96		.08	
Step 6 making environment safe (out of 2 lines)	2.0 (1.0-2.0)	2.0 (2.0-2.0)	2.0 (1.0-2.0)	2.0 (1.0-2.0)	2.84		.24	

## Discussion

Many patients who present to the ED with suicidality do not receive effective intervention [6]. EDs struggle with shortages in BH clinician availability and suicide-specific skills [15,16] and patients often describe their experiences of suicide-related care in the ED as negative [31]. ReachCare was developed to initiate an acceptable and feasible form of evidence-based care in the ED and support the patient in the high-risk period after discharge [17]. In this paper, we summarized initial implementation outcomes of the in-ED components of ReachCare across a variety of modalities. We found that patients rated the acceptability and appropriateness of the intervention highly in the context of other studies that have used the same measures [32-34], and there were no substantial differences across the various modalities. At just over 15 minutes, the self-administered, chatbot-style modality took less than half the time of the clinician modalities and did not require BH specialist input. The self-administered safety plans tended to have more content and the usability of the patient-facing tablet app was in the “very high” range. The safety plans that were created with ReachCare, whether self-administered with a chatbot or delivered by a clinician, were of high quality and completeness. Given that higher safety plan quality is associated with reduced risk of prospective suicidal behavior [35,36] and of subsequent psychiatric hospitalization [37], ReachCare has the potential to positively impact patient outcomes in a fully powered trial, even when it is implemented using self-administered modality.

Technology is becoming a key implementation strategy for BH interventions in health care settings [38]. Brief interventions for suicidality in the ED are recommended by the Joint Commission [39] and the American College of Emergency Physicians [40] but in practice have proven difficult to implement. In EDs, where there is a dearth of BH clinicians and the focus is on evaluation and disposition, engaging methods of self-administration may be one of the few ways to ensure that patients receive any intervention. Self-guided digital interventions for suicidality have been found to have a significant, if small, effect [41]. This study contributes to growing evidence that, when delivered through engaging digital platforms, self-guided versions of effective interventions can be efficient, feasible, and acceptable. For example, the tablet-based intervention “Jaspr” was associated with better care process and decreased distress in patients presenting with suicidality [42]. Similarly, a self-administered tablet-based safety planning website designed for use in the ED was associated with good usability and reduced suicidal intensity [43]. Finally, “Lock to Live,” is a promising tablet-based intervention for counseling on access to lethal means in the ED [44,45]. These encouraging results support the idea that self-administration could offer unique advantages in terms of timely, tailored, and scalable suicide intervention in resource-constrained EDs.

Like the broader population of people presenting with suicidality, our sample displayed a high prevalence of depression, anxiety disorder, trauma, and attention-deficit/hyperactivity disorder, as well as elevated levels of thwarted belongness and perceived burdensomeness. Given that those who are experiencing suicidality may be feeling particularly disconnected [46], there are ethical implications around simply handing a patient a tablet computer as a panacea to suicide-related care in the ED. The development of such technology-facilitated intervention should start with careful mapping of user needs from a variety of stakeholders and apply an iterative approach to testing and refinement [17,47]. Although self-administration holds promise as a way of scaling evidence-based interventions, programs that propagate loneliness and disempowerment in suicidal patients have the potential to do more harm than good. Any self-administered software for this population should be engaging but easy to navigate, foster a sense of connection as much as possible (for example by using videos and interaction), and anticipate and seek to ameliorate feelings of burdensomeness and disconnection. When done well, it is possible that technology could increase empowerment by introducing flexibility and choice to fit with patients’ timeline and needs [48,49]. Self-administered intervention in this population may be thoughtfully introduced by a clinician, such as a physician, nurse, or a medical assistant: the patient could be offered a choice, to complete a safety plan with a BH clinician (in-person or via telehealth if necessary) or for themselves if they prefer. A third option is a hybrid approach, where a patient initiates the safety plan using software and a BH clinician reviews it with them, ideally before discharge or as part of telephone follow-up. Intervention is ideally not limited to the ED but carries on out into the community in the form of caring contacts or outpatient engagement that can help to address the underlying drivers of the individual’s suicidality [50]. It is also important to note that technology is not a sufficient implementation strategy in itself: digital interventions require careful implementation planning, training, and support if they are to result in improved reach and sustainability [51].

Finally, our study led to some interesting observations around digitally supported clinician delivery. There appeared to be a slight trend toward lower acceptability of the telehealth clinician modality compared to the in-person clinician modality in our small trial. Larger trials that have compared telehealth to in-person delivery for a variety of psychosocial interventions have found little difference in satisfaction or effectiveness between the 2 modalities [52-54]. Moreover, telehealth evaluations can help to improve access and timeliness of mental health care in ED settings [55,56]. Further research is required to examine whether telemental health interventions specifically are less acceptable in an emergency medicine context.

This study has several limitations. Although the broader trial involved 3-month follow-up of clinical outcomes, the current analyses were cross-sectional only and our sample size was relatively small. In addition, the patients who were successfully enrolled tended to be younger, female, White, and non-Hispanic than those who were not enrolled. This may reflect the fact that the intervention was only available in English and trial enrollment required the patient to own a smartphone: additional user-centered research is needed to identify the needs of underserved populations and design systems that can adapt to their needs and preferences. For example, future research should explore Spanish-language adaptation of the intervention, as well as adaptations of the post-ED components that can be delivered without a smartphone. Notably, the majority of patients who screened positive for suicide risk during the trial were not eligible to participate because of medical or cognitive issues, which interfered with their ability to give informed consent: it remains to be seen what the reach of this intervention would be under naturalistic clinical conditions, where the capacity threshold for participation may be more flexible.

Our findings suggest that patients considered the ReachCare intervention in the ED to be acceptable and appropriate across a variety of modalities. Technology facilitation of evidence-based interventions in the ED holds much promise, with the caveat that we must take account of the particular and diverse needs of those in suicidal crisis in this setting. There are several potential directions for future research. These include possible adaptations for older patients, those whose preferred language is not English, and patients who do not own a smartphone. Other potential research questions include: which intervention modality patients or clinicians tend to select when given a choice; whether that choice is affected by patient characteristics such as age and medical condition; whether the selected modality affects longitudinal clinical outcomes; and rates of uptake under more naturalistic implementation conditions.

## Appendix

Multimedia Appendix 1 Patient characteristics at baseline (N=47) by study arm.

Multimedia Appendix 2 Characteristics of index ED visit (N=47) by arm.

Multimedia Appendix 3 CONSORT-eHEALTH checklist (V 1.6.1).

## Abbreviations

BH: behavioral health
ED: emergency department
ED-SAFE: Emergency Department Safety Assessment and Follow-up Evaluation
PSS: Patient Safety Screener

## Data Availability

Data are available in the National Institute of Mental Health Data Archive.
