# Supplementary material for: Comparing the Acceptability and Quality of Intervention Modalities for Suicidality in the Emergency Department: Randomized Feasibility Trial
Source: JMIR Ment Health. 2023 Oct 24;10:e49783. doi: 10.2196/49783 (PMC10630858; doi:10.2196/49783)
Supplement: Multimedia Appendix 1 [file mental_v10i1e49783_app1.docx]

Table S1. Patient characteristics at baseline (n=47) by study arm.

|  |  | **Arm 1**  **Self-administered** | | **Arm 2**  **Clinician**  **in-person** | | **Arm 3**  **Clinician telehealth** | |
| --- | --- | --- | --- | --- | --- | --- | --- |
| *Categorical variables* |  | **n** | **%** | **n** | **%** | **n** | **%** |
| Gender | Female | 10 | 62.5 | 7 | 46.7 | 7 | 43.8 |
|  | Male | 6 | 37.5 | 7 | 46.7 | 7 | 43.8 |
|  | Non-binary | 0 | 0 | 1 | 6.7 | 2 | 12.5 |
| Age | 18-24 years old |  |  |  |  |  |  |
|  | 25-34 years old |  |  |  |  |  |  |
|  | 35+ years old |  |  |  |  |  |  |
| Race (check all that apply) | White | 12 | 75.0% | 13 | 86.7% | 13 | 81.3% |
|  | Other | 2 | 12.5% | 1 | 6.7% | 2 | 12.5% |
|  | Black/African American | 2 | 12.5% | 1 | 6.7% | 1 | 6.3% |
|  | Asian | 1 | 6.3% | 0 | 0% | 1 | 6.3% |
|  | American Indian/Alaskan Native | 0 | 0% | 1 | 6.7% | 0 | 0% |
|  | Native Hawaiian/Pacific Islander | 0 | 0% | 0 | 0% | 0 | 0% |
| Ethnicity | Non-Hispanic/Latinx | 14 | 87.5% | 14 | 93.3% | 12 | 75.0% |
|  | Hispanic/Latinx | 2 | 12.5% | 1 | 6.7% | 4 | 25.0% |
| Insurance | Private | 8 | 50.0% | 6 | 40.0% | 9 | 56.3% |
|  | Public (e.g. Medicare, Medicaid) | 6 | 37.5% | 8 | 53.3% | 5 | 31.3% |
|  | None/self-pay | 2 | 12.5% | 1 | 6.7% | 2 | 12.5% |
| Psychiatric diagnoses (check all that apply) | Depressive disorder | 12 | 75.0% | 14 | 93.3% | 14 | 87.5% |
|  | Anxiety disorder | 9 | 56.3% | 13 | 86.7% | 16 | 100% |
|  | Trauma or stress-related | 7 | 43.8% | 10 | 66.7% | 7 | 43.8% |
|  | ADHD | 5 | 31.3% | 7 | 46.7% | 9 | 56.3% |
|  | Substance use disorder | 5 | 31.3% | 8 | 53.3% | 3 | 18.8% |
|  | Personality disorder | 4 | 25.0% | 5 | 33.3% | 3 | 18.8% |
|  | Eating disorder | 2 | 12.5% | 2 | 13.3% | 1 | 6.3% |
|  | Schizophrenia/psychotic disorder | 1 | 6.3% | 2 | 13.3% | 0 | 0% |
|  | Autism spectrum disorder | 1 | 6.3% | 0 | 0% | 1 | 6.3% |
|  | Other | 1 | 6.3% | 1 | 6.7% | 0 | 0% |
| Suicidal behavior | Past-week active ideation | 12 | 75.0% | 9 | 60.0% | 16 | 100% |
|  | Lifetime attempt | 11 | 68.8% | 11 | 73.3% | 8 | 50.0% |
|  | Current attempt | 4 | 25.0% | 1 | 6.7% | 2 | 12.5% |
| BH treatments received in past 3 months | Prescription medication | 10 | 62.5% | 12 | 80.0% | 15 | 93.8% |
|  | Individual therapy | 6 | 37.5% | 10 | 66.7% | 11 | 68.8% |
|  | Partial hospitalization/intensive outpatient | 7 | 43.8% | 5 | 33.3% | 5 | 31.3% |
|  | Behavioral health ED visit | 2 | 12.5% | 5 | 33.3% | 5 | 31.1% |
|  | Group support | 4 | 25.0% | 6 | 40.0% | 1 | 6.3% |
|  | Inpatient psychiatric hospitalization | 3 | 18,8% | 4 | 26.7% | 2 | 12.5% |
|  | Helpline support | 4 | 25.0% | 1 | 6.7% | 4 | 25.0% |
|  | Other | 3 | 18.8% | 2 | 13.3% | 4 | 25.0% |
|  | Family therapy/couples counseling | 1 | 6.3% | 0 | 0.0% | 3 | 18.8% |
| Lethal means | Access to medication | 12 | 75.0% | 13 | 86.7% | 15 | 93.8% |
|  | Access to firearm | 0 | 0.0% | 0 | 0.0% | 0 | 0.0% |
| Smartphone use in past week per day | > 3 hours a day | 14 | 87.5% | 10 | 66.7% | 11 | 68.8% |
|  | 30 mins – 3 hours | 1 | 6.3% | 3 | 20.0% | 5 | 31.3% |
|  | <30 mins a day | 1 | 6.3% | 0 | 0.0% | 0 | 0.0% |
|  | Rarely/never | 0 | 0.0% | 2 | 13.3% | 0 | 0.0% |
| *Continuous variables* |  |  |  |  |  |  |  |
| Smartphone self-efficacy | I can use smartphone technology if there was no one around to tell me what to do (max score 5) | 4.63 | 0.89 | 4.53 | 0.64 | 4.69 | 0.48 |
|  | I can use smartphone technology even if I have never used a similar technology before (max score 5) | 4.06 | 1.18 | 4.20 | 0.94 | 4.19 | 0.48 |
|  | I am confident that I can effectively open and use an app on my smartphone (max score 5) | 4.94 | 0.25 | 4.67 | 0.62 | 4.19 | 0.98 |
| Hypothesized mechanisms of action | INQ Perceived Burdensomeness subscale (Range 6-42) | 19.31 | 11.06 | 18.27 | 10.48 | 21.06 | 8.94 |
|  | INQ Thwarted Belongingness subscale (Range 9-63) | 31.38 | 13.88 | 30.93 | 15.43 | 39.00 | 8.91 |
|  | BAS-Drive subscale (range 4-16) | 12.38 | 2.50 | 10.33 | 3.37 | 10.75 | 2.91 |
|  | Suicide-related impulsivity (range 1-5) | 3.25 | 1.44 | 2.63 | 1.27 | 3.16 | 1.34 |

*INQ= Interpersonal Needs Questionnaire; BAS= Behavioral Activation Scale; ED= Emergency Department; BH= Behavioral Health*
