# Supplementary material for: Comparing the Acceptability and Quality of Intervention Modalities for Suicidality in the Emergency Department: Randomized Feasibility Trial
Source: JMIR Ment Health. 2023 Oct 24;10:e49783. doi: 10.2196/49783 (PMC10630858; doi:10.2196/49783)
Supplement: Multimedia Appendix 2 [file mental_v10i1e49783_app2.docx]

Table S2. Characteristics of index ED visit (n=47) by arm.

|  |  | **Arm 1**  **Self-administered** | | **Arm 2**  **Clinician**  **in-person** | | **Arm 3**  **Clinician telehealth** | |
| --- | --- | --- | --- | --- | --- | --- | --- |
|  |  | **n** | **%** | **n** | **%** | **n** | **%** |
| Enrollment location | Psychiatric ED | 11 | 6.8% | 9 | 60.0% | 10 | 62.5% |
|  | Medical ED | 5 | 31.3% | 6 | 40.0% | 6 | 37.5% |
| Chief complaint | Suicidality | 13 | 86.7% | 12 | 80.0% | 10 | 62.5% |
|  | Non-suicide psychiatric | 3 | 18.8% | 3 | 20.0% | 5 | 31.3% |
|  | Non-psychiatric | 0 | 0.0% | 0 | 0.0% | 1 | 6.3% |
| Involuntary behavioral health hold | Yes | 15 | 93.8% | 14 | 93.3% | 12 | 75.0% |
|  | No | 1 | 6.3% | 1 | 6.7% | 4 | 25.0% |
| Patient Safety Screener result (check all that apply) | Positive for active ideation in past 2 weeks | 14 | 87.5% | 14 | 93.3% | 15 | 93.8% |
|  | Positive for suicide attempt in past 6 mths | 5 | 31.3% | 4 | 26.7% | 6 | 37.5% |
| BH Evaluation | Yes | 13 | 81.3% | 14 | 93.3% | 13 | 81.3% |
|  | No | 3 | 18.8% | 1 | 6.7% | 3 | 18.8% |
| Referral to outpatient BH | Yes | 1 | 6.3% | 0 | 0.0% | 0 | 0.0% |
|  | No | 15 | 93.8% | 15 | 100.0% | 16 | 100.0% |
| Structured suicide assessment | Yes | 8 | 50.0% | 8 | 53.3% | 10 | 62.5% |
|  | No | 8 | 50.0% | 7 | 46.7% | 6 | 37.5% |
| Under observation | Yes | 15 | 93.8% | 15 | 100.0% | 15 | 93.8% |
|  | No | 1 | 6.3% | 0 | 0.0% | 1 | 6.3% |
| Disposition | Psychiatric admission/transfer | 12 | 74.9% | 7 | 46.7% | 10 | 62.5% |
|  | Home | 3 | 18.8% | 7 | 46.7% | 6 | 37.5% |
|  | Other/not documented | 1 | 6.3% | 1 | 6.7% | 0 | 0.00% |

*ED= Emergency Department; BH= Behavioral Health*
