# Supplementary material for: Comparing the Acceptability and Quality of Intervention Modalities for Suicidality in the Emergency Department: Randomized Feasibility Trial
Source: JMIR Ment Health. 2023 Oct 24;10:e49783. doi: 10.2196/49783 (PMC10630858; doi:10.2196/49783)
Supplement: Multimedia Appendix 3 [file mental_v10i1e49783_app3.pdf]

CONSORT-EHEALTH (V 1.6.1) - Submission/Publication Form

The CONSORT-EHEALTH checklist is intended for authors of randomized trials evaluating web-based and Internet-based applications/interventions, including mobile interventions, electronic games (incl multiplayer games), social media, certain telehealth applications, and other interactive and/or networked electronic applications. Some of the items (e.g. all subitems under item 5 - description of the intervention) may also be applicable for other study designs.

The goal of the CONSORT EHEALTH checklist and guideline is to be
a) a guide for reporting for authors of RCTs,
b) to form a basis for appraisal of an ehealth trial (in terms of validity)

CONSORT-EHEALTH items/subitems are MANDATORY reporting items for studies published in the Journal of Medical Internet Research and other journals / scientific societies endorsing the checklist.

Items numbered 1., 2., 3., 4a., 4b etc are original CONSORT or CONSORT-NPT (non-pharmacologic treatment) items.
Items with Roman numerals (i., ii, iii, iv etc.) are CONSORT-EHEALTH extensions/clarifications.

As the CONSORT-EHEALTH checklist is still considered in a formative stage, we would ask that you also RATE ON A SCALE OF 1-5 how important/useful you feel each item is FOR THE PURPOSE OF THE CHECKLIST and reporting guideline (optional).

Mandatory reporting items are marked with a red *.
In the textboxes, either copy & paste the relevant sections from your manuscript into this form - please include any quotes from your manuscript in QUOTATION MARKS,
or answer directly by providing additional information not in the manuscript, or elaborating on why the item was not relevant for this study.

YOUR ANSWERS WILL BE PUBLISHED AS A SUPPLEMENTARY FILE TO YOUR PUBLICATION IN JMIR AND ARE CONSIDERED PART OF YOUR PUBLICATION (IF ACCEPTED).
Please fill in these questions diligently. Information will not be copyedited, so please use proper spelling and grammar, use correct capitalization, and avoid abbreviations.

DO NOT FORGET TO SAVE AS PDF _AND_ CLICK THE SUBMIT BUTTON SO YOUR ANSWERS ARE IN OUR DATABASE !!!

Citation Suggestion (if you append the pdf as Appendix we suggest to cite this paper in the caption):
Eysenbach G, CONSORT-EHEALTH Group
CONSORT-EHEALTH: Improving and Standardizing Evaluation Reports of Web-based and Mobile Health Interventions
J Med Internet Res 2011;13(4):e126
URL: http://www.jmir.org/2011/4/e126/
doi: 10.2196/jmir.1923
PMID: 22209829

celinelarkin@gmail.com Switch account

Not shared

Draft saved

* Indicates required question

Your name *

First Last

Celine Larkin

Primary Affiliation (short), City, Country *

University of Toronto, Toronto, Canada

UMass Chan Medical School, Worcester, MA, U

Your e-mail address *

abc@gmail.com

celine.larkin@umassmed.edu

Title of your manuscript *

Provide the (draft) title of your manuscript.

Comparing the Acceptability and Quality of Intervention Modalities for Suicidality in the Emergency Department: Randomized Feasibility Trial

Name of your App/Software/Intervention *

If there is a short and a long/alternate name, write the short name first and add the long name in brackets.

ReachCare

Evaluated Version (if any)

e.g. "V1", "Release 2017-03-01", "Version 2.0.27913"

Version 1

Language(s) *

What language is the intervention/app in? If multiple languages are available, separate by comma (e.g. "English, French")

English

URL of your Intervention Website or App

e.g. a direct link to the mobile app on app in appstore (itunes, Google Play), or URL of the website. If the intervention is a DVD or hardware, you can also link to an Amazon page.

https://www.reachcareapp.org/login

URL of an image/screenshot (optional)

Your answer

Accessibility *

Can an enduser access the intervention presently?

- ○ access is free and open
- ◉ access only for special usergroups, not open
- ○ access is open to everyone, but requires payment/subscription/in-app purchases
- ○ app/intervention no longer accessible
- ○ Other:

Primary Medical Indication/Disease/Condition *

e.g. "Stress", "Diabetes", or define the target group in brackets after the condition, e.g. "Autism (Parents of children with)", "Alzheimers (Informal Caregivers of)"

Suicidality

Primary Outcomes measured in trial *

comma-separated list of primary outcomes reported in the trial

Acceptability, appropriateness, usability

Secondary/other outcomes

Are there any other outcomes the intervention is expected to affect?

Your answer

Recommended "Dose" *

What do the instructions for users say on how often the app should be used?

- ○ Approximately Daily
- ○ Approximately Weekly
- ○ Approximately Monthly
- ○ Approximately Yearly
- ◉ "as needed"
- ○ Other:

Approx. Percentage of Users (starters) still using the app as recommended after 3 months *

- ( ) unknown / not evaluated
- ( ) 0-10%
- ( ) 11-20%
- ( ) 21-30%
- ( ) 31-40%
- ( ) 41-50%
- ( ) 51-60%
- ( ) 61-70%
- ( ) 71%-80%
- ( ) 81-90%
- ( ) 91-100%
- (•) Other: This manuscript is not focused on longitudinal outcomes

Overall, was the app/intervention effective? *

- ○ yes: all primary outcomes were significantly better in intervention group vs control
- ○ partly: SOME primary outcomes were significantly better in intervention group vs control
- ○ no statistically significant difference between control and intervention
- ○ potentially harmful: control was significantly better than intervention in one or more outcomes
- ○ inconclusive: more research is needed
- ◉ Other: Clinical effectiveness was not being investigated in this manuscript

Article Preparation Status/Stage *

At which stage in your article preparation are you currently (at the time you fill in this form)

- ○ not submitted yet - in early draft status
- ○ not submitted yet - in late draft status, just before submission
- ○ submitted to a journal but not reviewed yet
- ○ submitted to a journal and after receiving initial reviewer comments
- ◉ submitted to a journal and accepted, but not published yet
- ○ published
- ○ Other:

Journal *

If you already know where you will submit this paper (or if it is already submitted), please provide the journal name (if it is not JMIR, provide the journal name under "other")

- ○ not submitted yet / unclear where I will submit this
- ○ Journal of Medical Internet Research (JMIR)
- ○ JMIR mHealth and UHealth
- ○ JMIR Serious Games
- ◉ JMIR Mental Health
- ○ JMIR Public Health
- ○ JMIR Formative Research
- ○ Other JMIR sister journal
- ○ Other:

Is this a full powered effectiveness trial or a pilot/feasibility trial? *

- ◉ Pilot/feasibility
- ○ Fully powered

Manuscript tracking number *

If this is a JMIR submission, please provide the manuscript tracking number under "other" (The ms tracking number can be found in the submission acknowledgement email, or when you login as author in JMIR. If the paper is already published in JMIR, then the ms tracking number is the four-digit number at the end of the DOI, to be found at the bottom of each published article in JMIR)

- ○ no ms number (yet) / not (yet) submitted to / published in JMIR
- ◉ Other: 49783

TITLE AND ABSTRACT

1a) TITLE: Identification as a randomized trial in the title

1a) Does your paper address CONSORT item 1a? *

I.e does the title contain the phrase "Randomized Controlled Trial"? (if not, explain the reason under "other")

- ◉ yes
- ○ Other:

1a-i) Identify the mode of delivery in the title

Identify the mode of delivery. Preferably use “web-based” and/or “mobile” and/or “electronic game” in the title. Avoid ambiguous terms like “online”, “virtual”, “interactive”. Use “Internet-based” only if Intervention includes non-web-based Internet components (e.g. email), use “computer-based” or “electronic” only if offline products are used. Use “virtual” only in the context of “virtual reality” (3-D worlds). Use “online” only in the context of “online support groups”. Complement or substitute product names with broader terms for the class of products (such as “mobile” or “smart phone” instead of “iphone”), especially if the application runs on different platforms.

	1	2	3	4	5	
subitem not at all important	○	○	○	○	○	essential

Does your paper address subitem 1a-i? *

Copy and paste relevant sections from manuscript title (include quotes in quotation marks "like this" to indicate direct quotes from your manuscript), or elaborate on this item by providing additional information not in the ms, or briefly explain why the item is not applicable/relevant for your study

Title is "Comparing the Acceptability and Quality of Intervention Modalities for Suicidality in the Emergency Department: Randomized Feasibility Trial"

1a-ii) Non-web-based components or important co-interventions in title

Mention non-web-based components or important co-interventions in title, if any (e.g., “with telephone support”).

	1	2	3	4	5	
subitem not at all important	○	○	○	○	○	essential

Does your paper address subitem 1a-ii?

Copy and paste relevant sections from manuscript title (include quotes in quotation marks "like this" to indicate direct quotes from your manuscript), or elaborate on this item by providing additional information not in the ms, or briefly explain why the item is not applicable/relevant for your study

Title is "Comparing the Acceptability and Quality of Intervention Modalities for Suicidality in the Emergency Department: Randomized Feasibility Trial"

1a-iii) Primary condition or target group in the title

Mention primary condition or target group in the title, if any (e.g., “for children with Type I Diabetes”) Example: A Web-based and Mobile Intervention with Telephone Support for Children with Type I Diabetes: Randomized Controlled Trial

	1	2	3	4	5	
subitem not at all important	○	○	○	○	○	essential

Does your paper address subitem 1a-iii? *

Copy and paste relevant sections from manuscript title (include quotes in quotation marks "like this" to indicate direct quotes from your manuscript), or elaborate on this item by providing additional information not in the ms, or briefly explain why the item is not applicable/relevant for your study

Title is "Comparing the Acceptability and Quality of Intervention Modalities for Suicidality in the Emergency Department: Randomized Feasibility Trial"

1b) ABSTRACT: Structured summary of trial design, methods, results, and conclusions

NPT extension: Description of experimental treatment, comparator, care providers, centers, and blinding status.

1b-i) Key features/functionalities/components of the intervention and comparator in the METHODS section of the ABSTRACT

Mention key features/functionalities/components of the intervention and comparator in the abstract. If possible, also mention theories and principles used for designing the site. Keep in mind the needs of systematic reviewers and indexers by including important synonyms. (Note: Only report in the abstract what the main paper is reporting. If this information is missing from the main body of text, consider adding it)

	1	2	3	4	5	
subitem not at all important	○	○	○	○	○	essential

Does your paper address subitem 1b-i? *

Copy and paste relevant sections from the manuscript abstract (include quotes in quotation marks "like this" to indicate direct quotes from your manuscript), or elaborate on this item by providing additional information not in the ms, or briefly explain why the item is not applicable/relevant for your study

Abstract says "Our aim was to test the effectiveness of the ReachCare intervention in its entirety through conducting a feasibility study with patients presenting with suicidality to the ED. We tested three different ways of receiving the ED-based components of ReachCare: (1) self-administered on the tablet application using a chatbot interface; (2) administered by an in-person clinician; or (3) administered by a telehealth clinician."

1b-ii) Level of human involvement in the METHODS section of the ABSTRACT

Clarify the level of human involvement in the abstract, e.g., use phrases like “fully automated” vs. “therapist/nurse/care provider/physician-assisted” (mention number and expertise of providers involved, if any). (Note: Only report in the abstract what the main paper is reporting. If this information is missing from the main body of text, consider adding it)

	1	2	3	4	5	
subitem not at all important	○	○	○	○	○	essential

Does your paper address subitem 1b-ii?

Copy and paste relevant sections from the manuscript abstract (include quotes in quotation marks "like this" to indicate direct quotes from your manuscript), or elaborate on this item by providing additional information not in the ms, or briefly explain why the item is not applicable/relevant for your study

Abstract describes "We tested three different ways of receiving the ED-based components of ReachCare: (1) self-administered on the tablet application using a chatbot interface; (2) administered by an in-person clinician; or (3) administered by a telehealth clinician."

1b-iii) Open vs. closed, web-based (self-assessment) vs. face-to-face assessments in the METHODS section of the ABSTRACT

Mention how participants were recruited (online vs. offline), e.g., from an open access website or from a clinic or a closed online user group (closed usergroup trial), and clarify if this was a purely web-based trial, or there were face-to-face components (as part of the intervention or for assessment). Clearly say if outcomes were self-assessed through questionnaires (as common in web-based trials). Note: In traditional offline trials, an open trial (open-label trial) is a type of clinical trial in which both the researchers and participants know which treatment is being administered. To avoid confusion, use “blinded” or “unblinded” to indicated the level of blinding instead of “open”, as “open” in web-based trials usually refers to “open access” (i.e. participants can self-enrol). (Note: Only report in the abstract what the main paper is reporting. If this information is missing from the main body of text, consider adding it)

	1	2	3	4	5	
subitem not at all important	○	○	○	○	○	essential

Does your paper address subitem 1b-iii?

Copy and paste relevant sections from the manuscript abstract (include quotes in quotation marks "like this" to indicate direct quotes from your manuscript), or elaborate on this item by providing additional information not in the ms, or briefly explain why the item is not applicable/relevant for your study

Participants conducted the research procedures in the emergency department: "47 ED patients who screened positive for suicide risk were randomly allocated to receive one of three delivery modalities of ReachCare in the ED"

1b-iv) RESULTS section in abstract must contain use data

Report number of participants enrolled/assessed in each group, the use/uptake of the intervention (e.g., attrition/adherence metrics, use over time, number of logins etc.), in addition to primary/secondary outcomes. (Note: Only report in the abstract what the main paper is reporting. If this information is missing from the main body of text, consider adding it)

	1	2	3	4	5	
subitem not at all important	○	○	○	○	○	essential

Does your paper address subitem 1b-iv?

Copy and paste relevant sections from the manuscript abstract (include quotes in quotation marks "like this" to indicate direct quotes from your manuscript), or elaborate on this item by providing additional information not in the ms, or briefly explain why the item is not applicable/relevant for your study

Your answer

1b-v) CONCLUSIONS/DISCUSSION in abstract for negative trials

Conclusions/Discussions in abstract for negative trials: Discuss the primary outcome - if the trial is negative (primary outcome not changed), and the intervention was not used, discuss whether negative results are attributable to lack of uptake and discuss reasons. (Note: Only report in the abstract what the main paper is reporting. If this information is missing from the main body of text, consider adding it)

	1	2	3	4	5	
subitem not at all important	○	○	○	○	○	essential

Does your paper address subitem 1b-v?

Copy and paste relevant sections from the manuscript abstract (include quotes in quotation marks "like this" to indicate direct quotes from your manuscript), or elaborate on this item by providing additional information not in the ms, or briefly explain why the item is not applicable/relevant for your study

N/A: not negative

Your answer must have a minimum of 25 characters.

INTRODUCTION

2a) In INTRODUCTION: Scientific background and explanation of rationale

2a-i) Problem and the type of system/solution

Describe the problem and the type of system/solution that is object of the study: intended as stand-alone intervention vs. incorporated in broader health care program? Intended for a particular patient population? Goals of the intervention, e.g., being more cost-effective to other interventions, replace or complement other solutions? (Note: Details about the intervention are provided in “Methods” under 5)

	1	2	3	4	5	
subitem not at all important	○	○	○	○	○	essential

Does your paper address subitem 2a-i? *

Copy and paste relevant sections from the manuscript (include quotes in quotation marks "like this" to indicate direct quotes from your manuscript), or elaborate on this item by providing additional information not in the ms, or briefly explain why the item is not applicable/relevant for your study

"After a brief decline in 2019-2020 [1, 2], suicide rates have again begun to rise in the United States [1]. Each year, there are over one million suicide-related visits to emergency departments I the United States [3], and EDs represent a feasible venue to screen and assess suicide risk [4, 5]. However, once risk is detected, many ED patients with suicide risk receive suboptimal care [6].
Several effective interventions have been developed for this patient population [7]. The Emergency Department Safety Assessment and Follow-up Evaluation (ED-SAFE) intervention [8], for example, featured paper-based self-administered safety planning and resource lists in the ED; this was followed by intensive telephone outreach to patients and a chosen significant other in the 12 months after discharge. The telephone counseling involved safety planning, outpatient treatment engagement, values clarification and life planning, and family communication and coordination. The ED-SAFE intervention was associated with a 30% reduction in the total number of suicide attempts in the intervention group compared to treatment-as-usual [8]. Despite its promise and cost-effectiveness [9], the ED-SAFE intervention was resource-intensive and difficult to sustain without research support. Such interventions routinely struggle to "cross the chasm" to become routine care [10].
Clinicians and leadership recognize that ED-based care for suicidality needs to improve but often face substantial constraints in effecting change [11; 12]. The dearth of effective care for suicidality in the ED can be attributed to a wide range of factors, including a hectic milieu focused on patient flow [13], a shortage of behavioral health providers [14], and a shortfall in suicide-related skills and confidence among ED clinicians [15, 16]. With these barriers in mind, we set out to adapt the ED-SAFE intervention to become "ReachCare," leveraging technology to make the intervention scalable in resource-constrained EDs, while avoiding a corresponding loss in fidelity.
We previously outlined the user-centered development and testing of the patient-facing technology aspects of the ReachCare intervention [17]. These technologies include a tablet-based application for use within the ED (that allows a patient to build a high-quality safety plan in the absence of a behavioral health clinician) and a patient- and family-facing mobile application (that houses the patient's safety plan and life plan, helpline resources, psychoeducation, distractions, and a behavioral health provider search engine). In that study, user-testing with current ED patients presenting with suicidality demonstrated high usability and acceptability of the technologies [17]. ReachCare is further supported by interoperable clinician-facing software that provides tips, call structure, and note-taking for clinicians, as well as allowing them to update and share patients' therapeutic tools during follow-up phone calls. This clinician-facing technology was designed to be used in-person or via telehealth. "

2a-ii) Scientific background, rationale: What is known about the (type of) system

Scientific background, rationale: What is known about the (type of) system that is the object of the study (be sure to discuss the use of similar systems for other conditions/diagnoses, if appropiate), motivation for the study, i.e. what are the reasons for and what is the context for this specific study, from which stakeholder viewpoint is the study performed, potential impact of findings [2]. Briefly justify the choice of the comparator.

	1	2	3	4	5	
subitem not at all important	○	○	○	○	○	essential

Does your paper address subitem 2a-ii? *

Copy and paste relevant sections from the manuscript (include quotes in quotation marks "like this" to indicate direct quotes from your manuscript), or elaborate on this item by providing additional information not in the ms, or briefly explain why the item is not applicable/relevant for your study

"After a brief decline in 2019-2020 [1, 2], suicide rates have again begun to rise in the United States [1]. Each year, there are over one million suicide-related visits to emergency departments I the United States [3], and EDs represent a feasible venue to screen and assess suicide risk [4, 5]. However, once risk is detected, many ED patients with suicide risk receive suboptimal care [6].
Several effective interventions have been developed for this patient population [7]. The Emergency Department Safety Assessment and Follow-up Evaluation (ED-SAFE) intervention [8], for example, featured paper-based self-administered safety planning and resource lists in the ED; this was followed by intensive telephone outreach to patients and a chosen significant other in the 12 months after discharge. The telephone counseling involved safety planning, outpatient treatment engagement, values clarification and life planning, and family communication and coordination. The ED-SAFE intervention was associated with a 30% reduction in the total number of suicide attempts in the intervention group compared to treatment-as-usual [8]. Despite its promise and cost-effectiveness [9], the ED-SAFE intervention was resource-intensive and difficult to sustain without research support. Such interventions routinely struggle to "cross the chasm" to become routine care [10].
Clinicians and leadership recognize that ED-based care for suicidality needs to improve but often face substantial constraints in effecting change [11; 12]. The dearth of effective care for suicidality in the ED can be attributed to a wide range of factors, including a hectic milieu focused on patient flow [13], a shortage of behavioral health providers [14], and a shortfall in suicide-related skills and confidence among ED clinicians [15, 16]. With these barriers in mind, we set out to adapt the ED-SAFE intervention to become "ReachCare," leveraging technology to make the intervention scalable in resource-constrained EDs, while avoiding a corresponding loss in fidelity.
We previously outlined the user-centered development and testing of the patient-facing technology aspects of the ReachCare intervention [17]. These technologies include a tablet-based application for use within the ED (that allows a patient to build a high-quality safety plan in the absence of a behavioral health clinician) and a patient- and family-facing mobile application (that houses the patient's safety plan and life plan, helpline resources, psychoeducation, distractions, and a behavioral health provider search engine). In that study, user-testing with current ED patients presenting with suicidality demonstrated high usability and acceptability of the technologies [17]. ReachCare is further supported by interoperable clinician-facing software that provides tips, call structure, and note-taking for clinicians, as well as allowing them to update and share patients' therapeutic tools during follow-up phone calls. This clinician-facing technology was designed to be used in-person or via telehealth.
[illegible]

To test the effectiveness of the ReachCare intervention in its entirety, we conducted a feasibility study with patients presenting with suicidality to the ED. We tested three different ways of receiving the ED-based components of ReachCare: (1) self-administered on the tablet application using a chatbot interface; (2) administered by an in-person clinician; or (3) administered by a telehealth clinician. Here, we present the findings from the ED-based portion of the feasibility trial, namely the acceptability and appropriateness of ReachCare in the ED overall, the quality and completeness of safety plans created in the ED, intervention duration, and a comparison of these measures across the three potential delivery modalities."

2b) In INTRODUCTION: Specific objectives or hypotheses

Does your paper address CONSORT subitem 2b? *

Copy and paste relevant sections from the manuscript (include quotes in quotation marks "like this" to indicate direct quotes from your manuscript), or elaborate on this item by providing additional information not in the ms, or briefly explain why the item is not applicable/relevant for your study

"To test the effectiveness of the ReachCare intervention in its entirety, we conducted a feasibility study with patients presenting with suicidality to the ED. We tested three different ways of receiving the ED-based components of ReachCare: (1) self-administered on the tablet application using a chatbot interface; (2) administered by an in-person clinician; or (3) administered by a telehealth clinician. Here, we present the findings from the ED-based portion of the feasibility trial, namely the acceptability and appropriateness of ReachCare in the ED overall, the quality and completeness of safety plans created in the ED, intervention duration, and a comparison of these measures across the three potential delivery modalities"

METHODS

3a) Description of trial design (such as parallel, factorial) including allocation ratio

Does your paper address CONSORT subitem 3a? *

Copy and paste relevant sections from the manuscript (include quotes in quotation marks "like this" to indicate direct quotes from your manuscript), or elaborate on this item by providing additional information not in the ms, or briefly explain why the item is not applicable/relevant for your study

"This study was a feasibility trial of the ReachCare intervention, in which eligible patients were randomized to receive ReachCare in the ED in one of three ways: (1) self-administered on the tablet application using a chatbot interface; (2) administered by an in-person clinician; or (3) administered by a telehealth clinician. Randomization was on a 1:1:1 ratio based on blocks of 6. Allocation was done after the baseline research interview to preserve blinding of the research assistant collecting the baseline demographic and clinical measures. Because the research assistant had to know the allocation in order to facilitate the intervention, blinding was not possible for the acceptability measures, which were completed after the intervention. The quality rating of the safety plans was completed after completion of the trial by an assessor blinded to allocation (see more detail under Measures)."

3b) Important changes to methods after trial commencement (such as eligibility criteria), with reasons

Does your paper address CONSORT subitem 3b? *

Copy and paste relevant sections from the manuscript (include quotes in quotation marks "like this" to indicate direct quotes from your manuscript), or elaborate on this item by providing additional information not in the ms, or briefly explain why the item is not applicable/relevant for your study

N/A

Your answer must have a minimum of 25 characters.

3b-i) Bug fixes, Downtimes, Content Changes

Bug fixes, Downtimes, Content Changes: ehealth systems are often dynamic systems. A description of changes to methods therefore also includes important changes made on the intervention or comparator during the trial (e.g., major bug fixes or changes in the functionality or content) (5-iii) and other “unexpected events” that may have influenced study design such as staff changes, system failures/downtimes, etc. [2].

	1	2	3	4	5	
subitem not at all important	○	○	○	○	○	essential

Does your paper address subitem 3b-i?

Copy and paste relevant sections from the manuscript (include quotes in quotation marks "like this" to indicate direct quotes from your manuscript), or elaborate on this item by providing additional information not in the ms, or briefly explain why the item is not applicable/relevant for your study

N/A: there was no downtime during enrollment and app use

4a) Eligibility criteria for participants

Does your paper address CONSORT subitem 4a? *

Copy and paste relevant sections from the manuscript (include quotes in quotation marks "like this" to indicate direct quotes from your manuscript), or elaborate on this item by providing additional information not in the ms, or briefly explain why the item is not applicable/relevant for your study

"This study took place in the ED of a large teaching hospital in the northeastern United States. Over a six-month period, we sought to enroll patients who presented to the ED and screened positive for suicidality on the Patient Safety Screener (PSS) [18]. The PSS is used universally and routinely at the enrollment ED: a positive screen is defined as active ideation in the past two weeks or a suicide attempt in the past six months. Patients were considered for inclusion if they (i) screened positive for suicidality on the PSS, (ii) were 18 years or older, (iii) were cognitively and medically able to consent and participate, (iv) owned a smartphone, and (v) had a stable mailing address to receive compensation. Patients were excluded if they were (i) a prisoner or in police custody, (ii) overly agitated or violent, or (iii) on precautions for infectious disease, such as COVID-19. "

4a-i) Computer / Internet literacy

Computer / Internet literacy is often an implicit “de facto” eligibility criterion - this should be explicitly clarified.

	1	2	3	4	5	
subitem not at all important	○	○	○	○	○	essential

Does your paper address subitem 4a-i?

Copy and paste relevant sections from the manuscript (include quotes in quotation marks "like this" to indicate direct quotes from your manuscript), or elaborate on this item by providing additional information not in the ms, or briefly explain why the item is not applicable/relevant for your study

"We assessed mobile technology self-efficacy by patient self-report using three items from Balapour et al. [20]. "

4a-ii) Open vs. closed, web-based vs. face-to-face assessments:

Open vs. closed, web-based vs. face-to-face assessments: Mention how participants were recruited (online vs. offline), e.g., from an open access website or from a clinic, and clarify if this was a purely web-based trial, or there were face-to-face components (as part of the intervention or for assessment), i.e., to what degree got the study team to know the participant. In online-only trials, clarify if participants were quasi-anonymous and whether having multiple identities was possible or whether technical or logistical measures (e.g., cookies, email confirmation, phone calls) were used to detect/prevent these.

	1	2	3	4	5	
subitem not at all important	○	○	○	○	○	essential

Does your paper address subitem 4a-ii? *

Copy and paste relevant sections from the manuscript (include quotes in quotation marks "like this" to indicate direct quotes from your manuscript), or elaborate on this item by providing additional information not in the ms, or briefly explain why the item is not applicable/relevant for your study

"Our measures included a variety of modalities, including patient self-report via interview, medical chart review, researcher measurement (of duration of intervention), and researcher rating (of safety plan quality). "

4a-iii) Information giving during recruitment

Information given during recruitment. Specify how participants were briefed for recruitment and in the informed consent procedures (e.g., publish the informed consent documentation as appendix, see also item X26), as this information may have an effect on user self-selection, user expectation and may also bias results.

	1	2	3	4	5	
subitem not at all important	○	○	○	○	○	essential

Does your paper address subitem 4a-iii?

Copy and paste relevant sections from the manuscript (include quotes in quotation marks "like this" to indicate direct quotes from your manuscript), or elaborate on this item by providing additional information not in the ms, or briefly explain why the item is not applicable/relevant for your study

"Potentially eligible patients were approached by a research assistant in the ED's medical or psychiatric area and were screened to confirm eligibility. The research assistant then explained the study: patients were informed as to what the study entailed, procedures to protect confidentiality and privacy, their right to withdraw at any time, and the potential risks and benefits of the study. If the patient was still interested in participating, the research assistant then administered a consent mini-quiz to test the patient's understanding of the information and invited them to read and sign an informed consent form."

4b) Settings and locations where the data were collected

Does your paper address CONSORT subitem 4b? *

Copy and paste relevant sections from the manuscript (include quotes in quotation marks "like this" to indicate direct quotes from your manuscript), or elaborate on this item by providing additional information not in the ms, or briefly explain why the item is not applicable/relevant for your study

"This study took place in the ED of a large teaching hospital in the northeastern United States"

4b-i) Report if outcomes were (self-)assessed through online questionnaires

Clearly report if outcomes were (self-)assessed through online questionnaires (as common in web-based trials) or otherwise.

	1	2	3	4	5	
subitem not at all important	○	○	○	○	○	essential

Does your paper address subitem 4b-i? *

Copy and paste relevant sections from the manuscript (include quotes in quotation marks "like this" to indicate direct quotes from your manuscript), or elaborate on this item by providing additional information not in the ms, or briefly explain why the item is not applicable/relevant for your study

N/A: participants completed a research interview

4b-ii) Report how institutional affiliations are displayed

Report how institutional affiliations are displayed to potential participants [on ehealth media], as affiliations with prestigious hospitals or universities may affect volunteer rates, use, and reactions with regards to an intervention.(Not a required item – describe only if this may bias results)

	1	2	3	4	5	
subitem not at all important	○	○	○	○	○	essential

Does your paper address subitem 4b-ii?

Copy and paste relevant sections from the manuscript (include quotes in quotation marks "like this" to indicate direct quotes from your manuscript), or elaborate on this item by providing additional information not in the ms, or briefly explain why the item is not applicable/relevant for your study

The institution, PI name, and programming company were included in the consent form

5) The interventions for each group with sufficient details to allow replication, including how and when they were actually administered

5-i) Mention names, credential, affiliations of the developers, sponsors, and owners

Mention names, credential, affiliations of the developers, sponsors, and owners [6] (if authors/evaluators are owners or developer of the software, this needs to be declared in a “Conflict of interest” section or mentioned elsewhere in the manuscript).

	1	2	3	4	5	
subitem not at all important	○	○	○	○	○	essential

Does your paper address subitem 5-i?

Copy and paste relevant sections from the manuscript (include quotes in quotation marks "like this" to indicate direct quotes from your manuscript), or elaborate on this item by providing additional information not in the ms, or briefly explain why the item is not applicable/relevant for your study

"The ED-based portion of the ReachCare intervention focuses on creating a 6-step safety plan [19]. The safety plan involves working with the patient to identify warning signs of an impending suicidal crisis, internal coping strategies, social supports and places for distraction, social supports for help, professionals for help, and making the environment safer by limiting access to lethal means. In the self-administered arm of the trial, patients engaged with a tablet-based application: they watched an introductory video (with the choice of watching a clinician, community member or animated character deliver the same information) and then proceeded to create a safety plan by interacting with a chatbot-style interface that presented cues and questions, to which the patient typed in personalized answers [17]. Once the safety plan had been created, the patient had the opportunity to review and edit it, before watching an outro video explaining the next steps in their care. In contrast, in the clinician in-person and telehealth arms, the patient completed safety planning with the clinician. In those arms, the clinician used the ReachCare clinician portal, which provides clinician-facing cues, suggested phrasing, and structure to introduce the intervention to the patient and to create a safety plan with them"

5-ii) Describe the history/development process

Describe the history/development process of the application and previous formative evaluations (e.g., focus groups, usability testing), as these will have an impact on adoption/use rates and help with interpreting results.

	1	2	3	4	5	
subitem not at all important	○	○	○	○	○	essential

Does your paper address subitem 5-ii?

Copy and paste relevant sections from the manuscript (include quotes in quotation marks "like this" to indicate direct quotes from your manuscript), or elaborate on this item by providing additional information not in the ms, or briefly explain why the item is not applicable/relevant for your study

"We previously outlined the user-centered development and testing of the patient-facing technology aspects of the ReachCare intervention [17]. These technologies include a tablet-based application for use within the ED (that allows a patient to build a high-quality safety plan in the absence of a behavioral health clinician) and a patient- and family-facing mobile application (that houses the patient's safety plan and life plan, helpline resources, psychoeducation, distractions, and a behavioral health provider search engine). In that study, user-testing with current ED patients presenting with suicidality demonstrated high usability and acceptability of the technologies [17]. ReachCare is further supported by interoperable clinician-facing software that provides tips, call structure, and note-taking for clinicians, as well as allowing them to update and share patients' therapeutic tools during follow-up phone calls. This clinician-facing technology was designed to be used in-person or via telehealth."

5-iii) Revisions and updating

Revisions and updating. Clearly mention the date and/or version number of the application/intervention (and comparator, if applicable) evaluated, or describe whether the intervention underwent major changes during the evaluation process, or whether the development and/or content was “frozen” during the trial. Describe dynamic components such as news feeds or changing content which may have an impact on the replicability of the intervention (for unexpected events see item 3b).

	1	2	3	4	5	
subitem not at all important	○	○	○	○	○	essential

Does your paper address subitem 5-iii?

Copy and paste relevant sections from the manuscript (include quotes in quotation marks "like this" to indicate direct quotes from your manuscript), or elaborate on this item by providing additional information not in the ms, or briefly explain why the item is not applicable/relevant for your study

Not applicable, no changes were made during the trial

5-iv) Quality assurance methods

Provide information on quality assurance methods to ensure accuracy and quality of information provided [1], if applicable.

	1	2	3	4	5	
subitem not at all important	○	○	○	○	○	essential

Does your paper address subitem 5-iv?

Copy and paste relevant sections from the manuscript (include quotes in quotation marks "like this" to indicate direct quotes from your manuscript), or elaborate on this item by providing additional information not in the ms, or briefly explain why the item is not applicable/relevant for your study

"The quality of the safety plans was rated using the Safety Planning Intervention Scoring Algorithm -Brief [27], which assesses whether the safety plan has content in each line and whether that content is sufficiently specific. Each line receives a rating of 0 (no content), 1 (poor content) or 2 (satisfactory content), which is summed to create a total quality score. This quality rating was completed by a researcher who was blinded to the allocation of the patient."

5-v) Ensure replicability by publishing the source code, and/or providing screenshots/screen-capture video, and/or providing flowcharts of the algorithms used

Ensure replicability by publishing the source code, and/or providing screenshots/screen-capture video, and/or providing flowcharts of the algorithms used. Replicability (i.e., other researchers should in principle be able to replicate the study) is a hallmark of scientific reporting.

	1	2	3	4	5	
subitem not at all important	○	○	○	○	○	essential

Does your paper address subitem 5-v?

Copy and paste relevant sections from the manuscript (include quotes in quotation marks "like this" to indicate direct quotes from your manuscript), or elaborate on this item by providing additional information not in the ms, or briefly explain why the item is not applicable/relevant for your study

"We previously outlined the user-centered development and testing of the patient-facing technology aspects of the ReachCare intervention [17]. These technologies include a tablet-based application for use within the ED (that allows a patient to build a high-quality safety plan in the absence of a behavioral health clinician) and a patient- and family-facing mobile application (that houses the patient's safety plan and life plan, helpline resources, psychoeducation, distractions, and a behavioral health provider search engine). In that study, user-testing with current ED patients presenting with suicidality demonstrated high usability and acceptability of the technologies [17]. "

5-vi) Digital preservation

Digital preservation: Provide the URL of the application, but as the intervention is likely to change or disappear over the course of the years; also make sure the intervention is archived (Internet Archive, webcitation.org, and/or publishing the source code or screenshots/videos alongside the article). As pages behind login screens cannot be archived, consider creating demo pages which are accessible without login.

	1	2	3	4	5	
subitem not at all important	○	○	○	○	○	essential

Does your paper address subitem 5-vi?

Copy and paste relevant sections from the manuscript (include quotes in quotation marks "like this" to indicate direct quotes from your manuscript), or elaborate on this item by providing additional information not in the ms, or briefly explain why the item is not applicable/relevant for your study

Screenshots have been published: ""We previously outlined the user-centered development and testing of the patient-facing technology aspects of the ReachCare intervention [17]. These technologies include a tablet-based application for use within the ED (that allows a patient to build a high-quality safety plan in the absence of a behavioral health clinician) and a patient- and family-facing mobile application (that houses the patient's safety plan and life plan, helpline resources, psychoeducation, distractions, and a behavioral health provider search engine). In that study, user-testing with current ED patients presenting with suicidality demonstrated high usability and acceptability of the technologies [17]. ReachCare is further supported by interoperable clinician-facing software that provides tips, call structure, and note-taking for clinicians, as well as allowing them to update and share patients' therapeutic tools during follow-up phone calls. This clinician-facing technology was designed to be used in-person or via telehealth.""

5-vii) Access

Access: Describe how participants accessed the application, in what setting/context, if they had to pay (or were paid) or not, whether they had to be a member of specific group. If known, describe how participants obtained “access to the platform and Internet” [1]. To ensure access for editors/reviewers/readers, consider to provide a “backdoor” login account or demo mode for reviewers/readers to explore the application (also important for archiving purposes, see vi).

	1	2	3	4	5	
subitem not at all important	○	○	○	○	○	essential

Does your paper address subitem 5-vii? *

Copy and paste relevant sections from the manuscript (include quotes in quotation marks "like this" to indicate direct quotes from your manuscript), or elaborate on this item by providing additional information not in the ms, or briefly explain why the item is not applicable/relevant for your study

"In the self-administered arm of the trial, patients engaged with a tablet-based application: they watched an introductory video (with the choice of watching a clinician, community member or animated character deliver the same information) and then proceeded to create a safety plan by interacting with a chatbot-style interface that presented cues and questions, to which the patient typed in personalized answers [17]. Once the safety plan had been created, the patient had the opportunity to review and edit it, before watching an outro video explaining the next steps in their care."

5-viii) Mode of delivery, features/functionalities/components of the intervention and comparator, and the theoretical framework

Describe mode of delivery, features/functionalities/components of the intervention and comparator, and the theoretical framework [6] used to design them (instructional strategy [1], behaviour change techniques, persuasive features, etc., see e.g., [7, 8] for terminology). This includes an in-depth description of the content (including where it is coming from and who developed it) [1],” whether [and how] it is tailored to individual circumstances and allows users to track their progress and receive feedback” [6]. This also includes a description of communication delivery channels and – if computer-mediated communication is a component – whether communication was synchronous or asynchronous [6]. It also includes information on presentation strategies [1], including page design principles, average amount of text on pages, presence of hyperlinks to other resources, etc. [1].

	1	2	3	4	5	
subitem not at all important	○	○	○	○	○	essential

Does your paper address subitem 5-viii? *

Copy and paste relevant sections from the manuscript (include quotes in quotation marks "like this" to indicate direct quotes from your manuscript), or elaborate on this item by providing additional information not in the ms, or briefly explain why the item is not applicable/relevant for your study

"The ED-based portion of the ReachCare intervention focuses on creating a 6-step safety plan [19]. The safety plan involves working with the patient to identify warning signs of an impending suicidal crisis, internal coping strategies, social supports and places for distraction, social supports for help, professionals for help, and making the environment safer by limiting access to lethal means. In the self-administered arm of the trial, patients engaged with a tablet-based application: they watched an introductory video (with the choice of watching a clinician, community member or animated character deliver the same information) and then proceeded to create a safety plan by interacting with a chatbot-style interface that presented cues and questions, to which the patient typed in personalized answers [17]. Once the safety plan had been created, the patient had the opportunity to review and edit it, before watching an outro video explaining the next steps in their care. In contrast, in the clinician in-person and telehealth arms, the patient completed safety planning with the clinician. In those arms, the clinician used the ReachCare clinician portal, which provides clinician-facing cues, suggested phrasing, and structure to introduce the intervention to the patient and to create a safety plan with them. "

5-ix) Describe use parameters

Describe use parameters (e.g., intended “doses” and optimal timing for use). Clarify what instructions or recommendations were given to the user, e.g., regarding timing, frequency, heaviness of use, if any, or was the intervention used ad libitum.

	1	2	3	4	5	
subitem not at all important	○	○	○	○	○	essential

Does your paper address subitem 5-ix?

Copy and paste relevant sections from the manuscript (include quotes in quotation marks "like this" to indicate direct quotes from your manuscript), or elaborate on this item by providing additional information not in the ms, or briefly explain why the item is not applicable/relevant for your study

The intervention was a one-time dose, duration is described in the paper, in Table 3

5-x) Clarify the level of human involvement

Clarify the level of human involvement (care providers or health professionals, also technical assistance) in the e-intervention or as co-intervention (detail number and expertise of professionals involved, if any, as well as “type of assistance offered, the timing and frequency of the support, how it is initiated, and the medium by which the assistance is delivered”. It may be necessary to distinguish between the level of human involvement required for the trial, and the level of human involvement required for a routine application outside of a RCT setting (discuss under item 21 – generalizability).

	1	2	3	4	5	
subitem not at all important	○	○	○	○	○	essential

Does your paper address subitem 5-x?

Copy and paste relevant sections from the manuscript (include quotes in quotation marks "like this" to indicate direct quotes from your manuscript), or elaborate on this item by providing additional information not in the ms, or briefly explain why the item is not applicable/relevant for your study

"In the self-administered arm of the trial, patients engaged with a tablet-based application: they watched an introductory video (with the choice of watching a clinician, community member or animated character deliver the same information) and then proceeded to create a safety plan by interacting with a chatbot-style interface that presented cues and questions, to which the patient typed in personalized answers [17]. Once the safety plan had been created, the patient had the opportunity to review and edit it, before watching an outro video explaining the next steps in their care. In contrast, in the clinician in-person and telehealth arms, the patient completed safety planning with the clinician. In those arms, the clinician used the ReachCare clinician portal, which provides clinician-facing cues, suggested phrasing, and structure to introduce the intervention to the patient and to create a safety plan with them. "

5-xi) Report any prompts/reminders used

Report any prompts/reminders used: Clarify if there were prompts (letters, emails, phone calls, SMS) to use the application, what triggered them, frequency etc. It may be necessary to distinguish between the level of prompts/reminders required for the trial, and the level of prompts/reminders for a routine application outside of a RCT setting (discuss under item 21 – generalizability).

	1	2	3	4	5	
subitem not at all important	○	○	○	○	○	essential

Does your paper address subitem 5-xi? *

Copy and paste relevant sections from the manuscript (include quotes in quotation marks "like this" to indicate direct quotes from your manuscript), or elaborate on this item by providing additional information not in the ms, or briefly explain why the item is not applicable/relevant for your study

Not applicable: this manuscript describes an intervention used one time in the emergency department

5-xii) Describe any co-interventions (incl. training/support)

Describe any co-interventions (incl. training/support): Clearly state any interventions that are provided in addition to the targeted eHealth intervention, as ehealth intervention may not be designed as stand-alone intervention. This includes training sessions and support [1]. It may be necessary to distinguish between the level of training required for the trial, and the level of training for a routine application outside of a RCT setting (discuss under item 21 – generalizability.

	1	2	3	4	5	
subitem not at all important	○	○	○	○	○	essential

Does your paper address subitem 5-xii? *

Copy and paste relevant sections from the manuscript (include quotes in quotation marks "like this" to indicate direct quotes from your manuscript), or elaborate on this item by providing additional information not in the ms, or briefly explain why the item is not applicable/relevant for your study

Not applicable: this manuscript describes an intervention used one time in the emergency department

6a) Completely defined pre-specified primary and secondary outcome measures, including how and when they were assessed

Does your paper address CONSORT subitem 6a? *

Copy and paste relevant sections from the manuscript (include quotes in quotation marks "like this" to indicate direct quotes from your manuscript), or elaborate on this item by providing additional information not in the ms, or briefly explain why the item is not applicable/relevant for your study

"Here, we present the findings from the ED-based portion of the feasibility trial, namely the acceptability and appropriateness of ReachCare in the ED overall, the quality and completeness of safety plans created in the ED, intervention duration, and a comparison of these measures across the three potential delivery modalities."

6a-i) Online questionnaires: describe if they were validated for online use and apply CHERRIES items to describe how the questionnaires were designed/deployed

If outcomes were obtained through online questionnaires, describe if they were validated for online use and apply CHERRIES items to describe how the questionnaires were designed/deployed [9].

	1	2	3	4	5	
subitem not at all important	○	○	○	○	○	essential

Does your paper address subitem 6a-i?

Copy and paste relevant sections from manuscript text

online questionnaires were not used

6a-ii) Describe whether and how “use” (including intensity of use/dosage) was defined/measured/monitored

Describe whether and how “use” (including intensity of use/dosage) was defined/measured/monitored (logins, logfile analysis, etc.). Use/adoption metrics are important process outcomes that should be reported in any ehealth trial.

	1	2	3	4	5	
subitem not at all important	○	○	○	○	○	essential

Does your paper address subitem 6a-ii?

Copy and paste relevant sections from manuscript text

Not applicable: this manuscript describes an intervention used one time in the emergency department

6a-iii) Describe whether, how, and when qualitative feedback from participants was obtained

Describe whether, how, and when qualitative feedback from participants was obtained (e.g., through emails, feedback forms, interviews, focus groups).

	1	2	3	4	5	
subitem not at all important	○	○	○	○	○	essential

Does your paper address subitem 6a-iii?

Copy and paste relevant sections from manuscript text

Not applicable: qualitative data were not obtained

6b) Any changes to trial outcomes after the trial commenced, with reasons

Does your paper address CONSORT subitem 6b? *

Copy and paste relevant sections from the manuscript (include quotes in quotation marks "like this" to indicate direct quotes from your manuscript), or elaborate on this item by providing additional information not in the ms, or briefly explain why the item is not applicable/relevant for your study

No changes to trial outcomes after the trial commenced

7a) How sample size was determined

NPT: When applicable, details of whether and how the clustering by care provides or centers was addressed

7a-i) Describe whether and how expected **attrition** was taken into account when calculating the sample size

Describe whether and how expected **attrition** was taken into account when calculating the sample size.

	1	2	3	4	5	
subitem not at all important	○	○	○	○	○	essential

Does your paper address subitem 7a-i?

Copy and paste relevant sections from manuscript title (include quotes in quotation marks "like this" to indicate direct quotes from your manuscript), or elaborate on this item by providing additional information not in the ms, or briefly explain why the item is not applicable/relevant for your study

Not applicable: study was about feasibility so power/sample size analyses were not required

7b) When applicable, explanation of any interim analyses and stopping guidelines

Does your paper address CONSORT subitem 7b? *

Copy and paste relevant sections from the manuscript (include quotes in quotation marks "like this" to indicate direct quotes from your manuscript), or elaborate on this item by providing additional information not in the ms, or briefly explain why the item is not applicable/relevant for your study

Not applicable: no interim analyses

8a) Method used to generate the random allocation sequence

NPT: When applicable, how care providers were allocated to each trial group

Does your paper address CONSORT subitem 8a? *

Copy and paste relevant sections from the manuscript (include quotes in quotation marks "like this" to indicate direct quotes from your manuscript), or elaborate on this item by providing additional information not in the ms, or briefly explain why the item is not applicable/relevant for your study

". Randomization was on a 1:1:1 ratio based on blocks of 6. Allocation was done after the baseline research interview to preserve blinding of the research assistant collecting the baseline demographic and clinical measures. "

8b) Type of randomisation; details of any restriction (such as blocking and block size)

Does your paper address CONSORT subitem 8b? *

Copy and paste relevant sections from the manuscript (include quotes in quotation marks "like this" to indicate direct quotes from your manuscript), or elaborate on this item by providing additional information not in the ms, or briefly explain why the item is not applicable/relevant for your study

"Randomization was on a 1:1:1 ratio based on blocks of 6. Allocation was done after the baseline research interview to preserve blinding of the research assistant collecting the baseline demographic and clinical measures"

9) Mechanism used to implement the random allocation sequence (such as sequentially numbered containers), describing any steps taken to conceal the sequence until interventions were assigned

Does your paper address CONSORT subitem 9? *

Copy and paste relevant sections from the manuscript (include quotes in quotation marks "like this" to indicate direct quotes from your manuscript), or elaborate on this item by providing additional information not in the ms, or briefly explain why the item is not applicable/relevant for your study

"Randomization was on a 1:1:1 ratio based on blocks of 6. Allocation was done after the baseline research interview to preserve blinding of the research assistant collecting the baseline demographic and clinical measures. "

10) Who generated the random allocation sequence, who enrolled participants, and who assigned participants to interventions

Does your paper address CONSORT subitem 10? *

Copy and paste relevant sections from the manuscript (include quotes in quotation marks "like this" to indicate direct quotes from your manuscript), or elaborate on this item by providing additional information not in the ms, or briefly explain why the item is not applicable/relevant for your study

"Allocation was done after the baseline research interview to preserve blinding of the research assistant collecting the baseline demographic and clinical measures. Because the research assistant had to know the allocation in order to facilitate the intervention, blinding was not possible for the acceptability measures, which were completed after the intervention. The quality rating of the safety plans was completed after completion of the trial by an assessor blinded to allocation (see more detail under Measures). "

11a) If done, who was blinded after assignment to interventions (for example, participants, care providers, those assessing outcomes) and how

NPT: Whether or not administering co-interventions were blinded to group assignment

11a-i) Specify who was blinded, and who wasn’t

Specify who was blinded, and who wasn’t. Usually, in web-based trials it is not possible to blind the participants [1, 3] (this should be clearly acknowledged), but it may be possible to blind outcome assessors, those doing data analysis or those administering co-interventions (if any).

	1	2	3	4	5	
subitem not at all important	○	○	○	○	○	essential

Does your paper address subitem 11a-i? *

Copy and paste relevant sections from the manuscript (include quotes in quotation marks "like this" to indicate direct quotes from your manuscript), or elaborate on this item by providing additional information not in the ms, or briefly explain why the item is not applicable/relevant for your study

"Allocation was done after the baseline research interview to preserve blinding of the research assistant collecting the baseline demographic and clinical measures. Because the research assistant had to know the allocation in order to facilitate the intervention, blinding was not possible for the acceptability measures, which were completed after the intervention. The quality rating of the safety plans was completed after completion of the trial by an assessor blinded to allocation (see more detail under Measures). "

11a-ii) Discuss e.g., whether participants knew which intervention was the "intervention of interest" and which one was the "comparator"

Informed consent procedures (4a-ii) can create biases and certain expectations - discuss e.g., whether participants knew which intervention was the "intervention of interest" and which one was the "comparator".

	1	2	3	4	5	
subitem not at all important	○	○	○	○	○	essential

Does your paper address subitem 11a-ii?

Copy and paste relevant sections from the manuscript (include quotes in quotation marks "like this" to indicate direct quotes from your manuscript), or elaborate on this item by providing additional information not in the ms, or briefly explain why the item is not applicable/relevant for your study

Not applicable, there was no "intervention of interest", all three interventions were of interest

11b) If relevant, description of the similarity of interventions

(this item is usually not relevant for ehealth trials as it refers to similarity of a placebo or sham intervention to a active medication/intervention)

Does your paper address CONSORT subitem 11b? *

Copy and paste relevant sections from the manuscript (include quotes in quotation marks "like this" to indicate direct quotes from your manuscript), or elaborate on this item by providing additional information not in the ms, or briefly explain why the item is not applicable/relevant for your study

"The ED-based portion of the ReachCare intervention focuses on creating a 6-step safety plan [19]. The safety plan involves working with the patient to identify warning signs of an impending suicidal crisis, internal coping strategies, social supports and places for distraction, social supports for help, professionals for help, and making the environment safer by limiting access to lethal means. In the self-administered arm of the trial, patients engaged with a tablet-based application: they watched an introductory video (with the choice of watching a clinician, community member or animated character deliver the same information) and then proceeded to create a safety plan by interacting with a chatbot-style interface that presented cues and questions, to which the patient typed in personalized answers [17]. Once the safety plan had been created, the patient had the opportunity to review and edit it, before watching an outro video explaining the next steps in their care. In contrast, in the clinician in-person and telehealth arms, the patient completed safety planning with the clinician. In those arms, the clinician used the ReachCare clinician portal, which provides clinician-facing cues, suggested phrasing, and structure to introduce the intervention to the patient and to create a safety plan with them. "

12a) Statistical methods used to compare groups for primary and secondary outcomes

NPT: When applicable, details of whether and how the clustering by care providers or centers was addressed

Does your paper address CONSORT subitem 12a? *

Copy and paste relevant sections from the manuscript (include quotes in quotation marks "like this" to indicate direct quotes from your manuscript), or elaborate on this item by providing additional information not in the ms, or briefly explain why the item is not applicable/relevant for your study

"For the first part of the results, we summarized patient and encounter characteristics using descriptive statistics. We then examined differences in implementation measures (mean duration, acceptability, appropriateness, and quality) across the three arms using one-way analyses of variance. Finally, we summarize completeness of the safety plans in each arm using descriptive statistics. All tests were two-sided with alpha set at p<0.05. Analyses were completed in Statistical Package for Social Sciences Version 28 [28]."

12a-i) Imputation techniques to deal with attrition / missing values

Imputation techniques to deal with attrition / missing values: Not all participants will use the intervention/comparator as intended and attrition is typically high in ehealth trials. Specify how participants who did not use the application or dropped out from the trial were treated in the statistical analysis (a complete case analysis is strongly discouraged, and simple imputation techniques such as LOCF may also be problematic [4]).

	1	2	3	4	5	
subitem not at all important	○	○	○	○	○	essential

Does your paper address subitem 12a-i? *

Copy and paste relevant sections from the manuscript (include quotes in quotation marks "like this" to indicate direct quotes from your manuscript), or elaborate on this item by providing additional information not in the ms, or briefly explain why the item is not applicable/relevant for your study

Not applicable, attrition was not relevant as outcomes were measured immediately after intervention

12b) Methods for additional analyses, such as subgroup analyses and adjusted analyses

Does your paper address CONSORT subitem 12b? *

Copy and paste relevant sections from the manuscript (include quotes in quotation marks "like this" to indicate direct quotes from your manuscript), or elaborate on this item by providing additional information not in the ms, or briefly explain why the item is not applicable/relevant for your study

Not applicable, no subgroup analyses were conducted

X26) REB/IRB Approval and Ethical Considerations [recommended as subheading under "Methods"] (not a CONSORT item)

X26-i) Comment on ethics committee approval

	1	2	3	4	5	
subitem not at all important	○	○	○	○	○	essential

Does your paper address subitem X26-i?

Copy and paste relevant sections from the manuscript (include quotes in quotation marks "like this" to indicate direct quotes from your manuscript), or elaborate on this item by providing additional information not in the ms, or briefly explain why the item is not applicable/relevant for your study

"This study was approved by the Institutional Review Board of the University of Massachusetts Chan Medical School on 5/19/2020, under docket number H00020238. "

x26-ii) Outline informed consent procedures

Outline informed consent procedures e.g., if consent was obtained offline or online (how? Checkbox, etc.?), and what information was provided (see 4a-ii). See [6] for some items to be included in informed consent documents.

	1	2	3	4	5	
subitem not at all important	○	○	○	○	○	essential

Does your paper address subitem X26-ii?

Copy and paste relevant sections from the manuscript (include quotes in quotation marks "like this" to indicate direct quotes from your manuscript), or elaborate on this item by providing additional information not in the ms, or briefly explain why the item is not applicable/relevant for your study

"Potentially eligible patients were approached by a research assistant in the ED's medical or psychiatric area and were screened to confirm eligibility. The research assistant then explained the study: patients were informed as to what the study entailed, procedures to protect confidentiality and privacy, their right to withdraw at any time, and the potential risks and benefits of the study. If the patient was still interested in participating, the research assistant then administered a consent mini-quiz to test the patient's understanding of the information and invited them to read and sign an informed consent form. "

X26-iii) Safety and security procedures

Safety and security procedures, incl. privacy considerations, and any steps taken to reduce the likelihood or detection of harm (e.g., education and training, availability of a hotline)

	1	2	3	4	5	
subitem not at all important	○	○	○	○	○	essential

Does your paper address subitem X26-iii?

Copy and paste relevant sections from the manuscript (include quotes in quotation marks "like this" to indicate direct quotes from your manuscript), or elaborate on this item by providing additional information not in the ms, or briefly explain why the item is not applicable/relevant for your study

"Potentially eligible patients were approached by a research assistant in the ED’s medical or psychiatric area and were screened to confirm eligibility. The research assistant then explained the study: patients were informed as to what the study entailed, procedures to protect confidentiality and privacy, their right to withdraw at any time, and the potential risks and benefits of the study. If the patient was still interested in participating, the research assistant then administered a consent mini-quiz to test the patient’s understanding of the information and invited them to read and sign an informed consent form."

RESULTS

13a) For each group, the numbers of participants who were randomly assigned, received intended treatment, and were analysed for the primary outcome

NPT: The number of care providers or centers performing the intervention in each group and the number of patients treated by each care provider in each center

Does your paper address CONSORT subitem 13a? *

Copy and paste relevant sections from the manuscript (include quotes in quotation marks "like this" to indicate direct quotes from your manuscript), or elaborate on this item by providing additional information not in the ms, or briefly explain why the item is not applicable/relevant for your study

"47 agreed to participate and 46 completed the intervention and all research measures". The number of participants in each arms is described in Table 3

13b) For each group, losses and exclusions after randomisation, together with reasons

Does your paper address CONSORT subitem 13b? (NOTE: Preferably, this is shown in a CONSORT flow diagram) *

Copy and paste relevant sections from the manuscript (include quotes in quotation marks "like this" to indicate direct quotes from your manuscript), or elaborate on this item by providing additional information not in the ms, or briefly explain why the item is not applicable/relevant for your study

These are outlined in the consort diagram

13b-i) Attrition diagram

Strongly recommended: An attrition diagram (e.g., proportion of participants still logging in or using the intervention/comparator in each group plotted over time, similar to a survival curve) or other figures or tables demonstrating usage/dose/engagement.

	1	2	3	4	5	
subitem not at all important	○	○	○	○	○	essential

Does your paper address subitem 13b-i?

Copy and paste relevant sections from the manuscript or cite the figure number if applicable (include quotes in quotation marks "like this" to indicate direct quotes from your manuscript), or elaborate on this item by providing additional information not in the ms, or briefly explain why the item is not applicable/relevant for your study

Not applicable, attrition was not relevant as outcomes were measured immediately after intervention

14a) Dates defining the periods of recruitment and follow-up

Does your paper address CONSORT subitem 14a? *

Copy and paste relevant sections from the manuscript (include quotes in quotation marks "like this" to indicate direct quotes from your manuscript), or elaborate on this item by providing additional information not in the ms, or briefly explain why the item is not applicable/relevant for your study

Not applicable, outcomes were measured immediately after intervention

14a-i) Indicate if critical “secular events” fell into the study period

Indicate if critical “secular events” fell into the study period, e.g., significant changes in Internet resources available or “changes in computer hardware or Internet delivery resources”

	1	2	3	4	5	
subitem not at all important	○	○	○	○	○	essential

Does your paper address subitem 14a-i?

Copy and paste relevant sections from the manuscript (include quotes in quotation marks "like this" to indicate direct quotes from your manuscript), or elaborate on this item by providing additional information not in the ms, or briefly explain why the item is not applicable/relevant for your study

Not applicable, outcomes were measured immediately after intervention

14b) Why the trial ended or was stopped (early)

Does your paper address CONSORT subitem 14b? *

Copy and paste relevant sections from the manuscript (include quotes in quotation marks "like this" to indicate direct quotes from your manuscript), or elaborate on this item by providing additional information not in the ms, or briefly explain why the item is not applicable/relevant for your study

Not applicable, trial was not stopped early

15) A table showing baseline demographic and clinical characteristics for each group

NPT: When applicable, a description of care providers (case volume, qualification, expertise, etc.) and centers (volume) in each group

Does your paper address CONSORT subitem 15? *

Copy and paste relevant sections from the manuscript (include quotes in quotation marks "like this" to indicate direct quotes from your manuscript), or elaborate on this item by providing additional information not in the ms, or briefly explain why the item is not applicable/relevant for your study

See Table 1 for demographic details

15-i) Report demographics associated with digital divide issues

In ehealth trials it is particularly important to report demographics associated with digital divide issues, such as age, education, gender, social-economic status, computer/Internet/ehealth literacy of the participants, if known.

	1	2	3	4	5	
subitem not at all important	○	○	○	○	○	essential

Does your paper address subitem 15-i? *

Copy and paste relevant sections from the manuscript (include quotes in quotation marks "like this" to indicate direct quotes from your manuscript), or elaborate on this item by providing additional information not in the ms, or briefly explain why the item is not applicable/relevant for your study

See Table 1 for demographic details

16) For each group, number of participants (denominator) included in each analysis and whether the analysis was by original assigned groups

16-i) Report multiple “denominators” and provide definitions

Report multiple “denominators” and provide definitions: Report N’s (and effect sizes) “across a range of study participation [and use] thresholds” [1], e.g., N exposed, N consented, N used more than x times, N used more than y weeks, N participants “used” the intervention/comparator at specific pre-defined time points of interest (in absolute and relative numbers per group). Always clearly define “use” of the intervention.

	1	2	3	4	5	
subitem not at all important	○	○	○	○	○	essential

Does your paper address subitem 16-i? *

Copy and paste relevant sections from the manuscript (include quotes in quotation marks "like this" to indicate direct quotes from your manuscript), or elaborate on this item by providing additional information not in the ms, or briefly explain why the item is not applicable/relevant for your study

Outlined in each of the tables

16-ii) Primary analysis should be intent-to-treat

Primary analysis should be intent-to-treat, secondary analyses could include comparing only “users”, with the appropriate caveats that this is no longer a randomized sample (see 18-i).

	1	2	3	4	5	
subitem not at all important	○	○	○	○	○	essential

Does your paper address subitem 16-ii?

Copy and paste relevant sections from the manuscript (include quotes in quotation marks "like this" to indicate direct quotes from your manuscript), or elaborate on this item by providing additional information not in the ms, or briefly explain why the item is not applicable/relevant for your study

Not applicable, outcomes were measured immediately after intervention and participants did not "switch" arms

17a) For each primary and secondary outcome, results for each group, and the estimated effect size and its precision (such as 95% confidence interval)

Does your paper address CONSORT subitem 17a? *

Copy and paste relevant sections from the manuscript (include quotes in quotation marks "like this" to indicate direct quotes from your manuscript), or elaborate on this item by providing additional information not in the ms, or briefly explain why the item is not applicable/relevant for your study

This is provided in Table 3

17a-i) Presentation of process outcomes such as metrics of use and intensity of use

In addition to primary/secondary (clinical) outcomes, the presentation of process outcomes such as metrics of use and intensity of use (dose, exposure) and their operational definitions is critical. This does not only refer to metrics of attrition (13-b) (often a binary variable), but also to more continuous exposure metrics such as "average session length". These must be accompanied by a technical description how a metric like a "session" is defined (e.g., timeout after idle time) [1] (report under item 6a).

	1	2	3	4	5	
subitem not at all important	○	○	○	○	○	essential

Does your paper address subitem 17a-i?

Copy and paste relevant sections from the manuscript (include quotes in quotation marks "like this" to indicate direct quotes from your manuscript), or elaborate on this item by providing additional information not in the ms, or briefly explain why the item is not applicable/relevant for your study

We report duration of use in Table 3

17b) For binary outcomes, presentation of both absolute and relative effect sizes is recommended

Does your paper address CONSORT subitem 17b? *

Copy and paste relevant sections from the manuscript (include quotes in quotation marks "like this" to indicate direct quotes from your manuscript), or elaborate on this item by providing additional information not in the ms, or briefly explain why the item is not applicable/relevant for your study

Not applicable, binary outcomes not used

18) Results of any other analyses performed, including subgroup analyses and adjusted analyses, distinguishing pre-specified from exploratory

Does your paper address CONSORT subitem 18? *

Copy and paste relevant sections from the manuscript (include quotes in quotation marks "like this" to indicate direct quotes from your manuscript), or elaborate on this item by providing additional information not in the ms, or briefly explain why the item is not applicable/relevant for your study

Not applicable, subgroup analyses not done

18-i) Subgroup analysis of comparing only users

A subgroup analysis of comparing only users is not uncommon in ehealth trials, but if done, it must be stressed that this is a self-selected sample and no longer an unbiased sample from a randomized trial (see 16-iii).

	1	2	3	4	5	
subitem not at all important	○	○	○	○	○	essential

Does your paper address subitem 18-i?

Copy and paste relevant sections from the manuscript (include quotes in quotation marks "like this" to indicate direct quotes from your manuscript), or elaborate on this item by providing additional information not in the ms, or briefly explain why the item is not applicable/relevant for your study

Not applicable, subgroup analyses of only users not done

19) All important harms or unintended effects in each group

(for specific guidance see CONSORT for harms)

Does your paper address CONSORT subitem 19? *

Copy and paste relevant sections from the manuscript (include quotes in quotation marks "like this" to indicate direct quotes from your manuscript), or elaborate on this item by providing additional information not in the ms, or briefly explain why the item is not applicable/relevant for your study

No important harms or unintended effects occurred

19-i) Include privacy breaches, technical problems

Include privacy breaches, technical problems. This does not only include physical “harm” to participants, but also incidents such as perceived or real privacy breaches [1], technical problems, and other unexpected/unintended incidents. “Unintended effects” also includes unintended positive effects [2].

	1	2	3	4	5	
subitem not at all important	○	○	○	○	○	essential

Does your paper address subitem 19-i?

Copy and paste relevant sections from the manuscript (include quotes in quotation marks "like this" to indicate direct quotes from your manuscript), or elaborate on this item by providing additional information not in the ms, or briefly explain why the item is not applicable/relevant for your study

No breaches or technical problems occurred

19-ii) Include qualitative feedback from participants or observations from staff/researchers

Include qualitative feedback from participants or observations from staff/researchers, if available, on strengths and shortcomings of the application, especially if they point to unintended/unexpected effects or uses. This includes (if available) reasons for why people did or did not use the application as intended by the developers.

	1	2	3	4	5	
subitem not at all important	○	○	○	○	○	essential

Does your paper address subitem 19-ii?

Copy and paste relevant sections from the manuscript (include quotes in quotation marks "like this" to indicate direct quotes from your manuscript), or elaborate on this item by providing additional information not in the ms, or briefly explain why the item is not applicable/relevant for your study

Not applicable- qualitative feedback was not collected

DISCUSSION

22) Interpretation consistent with results, balancing benefits and harms, and considering other relevant evidence

NPT: In addition, take into account the choice of the comparator, lack of or partial blinding, and unequal expertise of care providers or centers in each group

22-i) Restate study questions and summarize the answers suggested by the data, starting with primary outcomes and process outcomes (use)

Restate study questions and summarize the answers suggested by the data, starting with primary outcomes and process outcomes (use).

	1	2	3	4	5	
subitem not at all important	○	○	○	○	○	essential

Does your paper address subitem 22-i? *

Copy and paste relevant sections from the manuscript (include quotes in quotation marks "like this" to indicate direct quotes from your manuscript), or elaborate on this item by providing additional information not in the ms, or briefly explain why the item is not applicable/relevant for your study

"Many patients who present to the ED with suicidality do not receive effective intervention [6]. EDs struggle with shortages in behavioral health clinician availability and suicide-specific skills [15, 16] and patients often describe their experiences of suicide-related care in the ED as negative [29]. ReachCare was developed to initiate an acceptable and feasible form of evidence-based care in the ED and support the patient in the high-risk period after discharge [17]. In the current manuscript, we summarized initial implementation outcomes of the in-ED components of ReachCare across a variety of modalities. We found that patients rated the acceptability and appropriateness of the intervention highly, and there were no substantial differences across the various modalities. At just over 15 minutes, the self-administered, chatbot-style modality took less than half the time of the clinician modalities and did not require behavioral health specialist input. The self-administered safety plans tended to have more content and the usability of the patient-facing tablet application was in the “very high” range. The safety plans that were created with ReachCare, whether self-administered with a chatbot or delivered by a clinician, were of high quality and completeness. "

22-ii) Highlight unanswered new questions, suggest future research

Highlight unanswered new questions, suggest future research.

	1	2	3	4	5	
subitem not at all important	○	○	○	○	○	essential

Does your paper address subitem 22-ii?

Copy and paste relevant sections from the manuscript (include quotes in quotation marks "like this" to indicate direct quotes from your manuscript), or elaborate on this item by providing additional information not in the ms, or briefly explain why the item is not applicable/relevant for your study

"Notably, the majority of patients who screened positive for suicide risk during the trial were not eligible to participate because of medical or cognitive issues: it remains to be seen what the reach of this intervention would be under naturalistic clinical conditions. "

20) Trial limitations, addressing sources of potential bias, imprecision, and, if relevant, multiplicity of analyses

20-i) Typical limitations in ehealth trials

Typical limitations in ehealth trials: Participants in ehealth trials are rarely blinded. Ehealth trials often look at a multiplicity of outcomes, increasing risk for a Type I error. Discuss biases due to non-use of the intervention/usability issues, biases through informed consent procedures, unexpected events.

	1	2	3	4	5	
subitem not at all important	○	○	○	○	○	essential

Does your paper address subitem 20-i? *

Copy and paste relevant sections from the manuscript (include quotes in quotation marks "like this" to indicate direct quotes from your manuscript), or elaborate on this item by providing additional information not in the ms, or briefly explain why the item is not applicable/relevant for your study

"The current study has several limitations. Although the broader trial involved three-month follow-up of clinical outcomes, the current analyses were cross-sectional only and our sample size was relatively small. In addition, the patients who were successfully enrolled skewed younger, female, White, and non-Hispanic than those who were not enrolled. This may reflect the fact that the intervention was only available in English and trial enrollment required the patient to own a smartphone: more user-centered research is needed to identify the needs of underserved populations and design systems that can adapt to their needs and preferences. Notably, the majority of patients who screened positive for suicide risk during the trial were not eligible to participate because of medical or cognitive issues: it remains to be seen what the reach of this intervention would be under naturalistic clinical conditions. "

21) Generalisability (external validity, applicability) of the trial findings

NPT: External validity of the trial findings according to the intervention, comparators, patients, and care providers or centers involved in the trial

21-i) Generalizability to other populations

Generalizability to other populations: In particular, discuss generalizability to a general Internet population, outside of a RCT setting, and general patient population, including applicability of the study results for other organizations

	1	2	3	4	5	
subitem not at all important	○	○	○	○	○	essential

Does your paper address subitem 21-i?

Copy and paste relevant sections from the manuscript (include quotes in quotation marks "like this" to indicate direct quotes from your manuscript), or elaborate on this item by providing additional information not in the ms, or briefly explain why the item is not applicable/relevant for your study

"The patients who were successfully enrolled skewed younger, female, White, and non-Hispanic than those who were not enrolled. This may reflect the fact that the intervention was only available in English and trial enrollment required the patient to own a smartphone: more user-centered research is needed to identify the needs of underserved populations and design systems that can adapt to their needs and preferences. Notably, the majority of patients who screened positive for suicide risk during the trial were not eligible to participate because of medical or cognitive issues"

21-ii) Discuss if there were elements in the RCT that would be different in a routine application setting

Discuss if there were elements in the RCT that would be different in a routine application setting (e.g., prompts/reminders, more human involvement, training sessions or other co-interventions) and what impact the omission of these elements could have on use, adoption, or outcomes if the intervention is applied outside of a RCT setting.

	1	2	3	4	5	
subitem not at all important	O	O	O	O	O	essential

Does your paper address subitem 21-ii?

Copy and paste relevant sections from the manuscript (include quotes in quotation marks "like this" to indicate direct quotes from your manuscript), or elaborate on this item by providing additional information not in the ms, or briefly explain why the item is not applicable/relevant for your study

"Self-administered intervention in this population may be thoughtfully introduced by a clinician, like a physician, nurse, or a medical assistant: the patient could be offered a choice, to complete a safety plan with a behavioral health clinician (in-person or via telehealth if necessary) or for themselves if they prefer. A third option is a hybrid approach, where a patient initiates the safety plan using software and a behavioral health clinician reviews it with them, ideally before discharge or as part of telephone follow-up."

OTHER INFORMATION

23) Registration number and name of trial registry

Does your paper address CONSORT subitem 23? *

Copy and paste relevant sections from the manuscript (include quotes in quotation marks "like this" to indicate direct quotes from your manuscript), or elaborate on this item by providing additional information not in the ms, or briefly explain why the item is not applicable/relevant for your study

NCT04720911

Your answer must have a minimum of 25 characters.

24) Where the full trial protocol can be accessed, if available

Does your paper address CONSORT subitem 24? *

Cite a Multimedia Appendix, other reference, or copy and paste relevant sections from the manuscript (include quotes in quotation marks "like this" to indicate direct quotes from your manuscript), or elaborate on this item by providing additional information not in the ms, or briefly explain why the item is not applicable/relevant for your study

Full trial protocol is not available but many details are documented at https://classic.clinicaltrials.gov/ct2/show/NCT04720911

25) Sources of funding and other support (such as supply of drugs), role of funders

Does your paper address CONSORT subitem 25? *

Copy and paste relevant sections from the manuscript (include quotes in quotation marks "like this" to indicate direct quotes from your manuscript), or elaborate on this item by providing additional information not in the ms, or briefly explain why the item is not applicable/relevant for your study

This research was supported by the National Institute of Mental Health of the National Institutes of Health under award number R34MH123578. The content is the sole responsibility of the authors and does not necessarily represent the official views of the National Institutes of Health.

X27) Conflicts of Interest (not a CONSORT item)

X27-i) State the relation of the study team towards the system being evaluated

In addition to the usual declaration of interests (financial or otherwise), also state the relation of the study team towards the system being evaluated, i.e., state if the authors/evaluators are distinct from or identical with the developers/sponsors of the intervention.

	1	2	3	4	5	
subitem not at all important	○	○	○	○	○	essential

Does your paper address subitem X27-i?

Copy and paste relevant sections from the manuscript (include quotes in quotation marks "like this" to indicate direct quotes from your manuscript), or elaborate on this item by providing additional information not in the ms, or briefly explain why the item is not applicable/relevant for your study

Authors/evaluators are the developers of the intervention.

About the CONSORT EHEALTH checklist

As a result of using this checklist, did you make changes in your manuscript? *

- ◯ yes, major changes
- ◉ yes, minor changes
- ◯ no

What were the most important changes you made as a result of using this checklist?

Clarifying that the authors/evaluators are the developers of the intervention.

How much time did you spend on going through the checklist INCLUDING making changes in your manuscript *

60 minutes................

As a result of using this checklist, do you think your manuscript has improved? *

- ○ yes
- ○ no
- ◉ Other: This form was not user-friendly and felt burdensome, some of the iten

Would you like to become involved in the CONSORT EHEALTH group?

This would involve for example becoming involved in participating in a workshop and writing an "Explanation and Elaboration" document

- ○ yes
- ◉ no
- ○ Other:

Clear selection

Any other comments or questions on CONSORT EHEALTH

Your answer

STOP - Save this form as PDF before you click submit

To generate a record that you filled in this form, we recommend to generate a PDF of this page (on a Mac, simply select "print" and then select "print as PDF") before you submit it.

When you submit your (revised) paper to JMIR, please upload the PDF as supplementary file.

Don't worry if some text in the textboxes is cut off, as we still have the complete information in our database. Thank you!

Final step: Click submit !

Click submit so we have your answers in our database!

Submit Clear form

Never submit passwords through Google Forms.

This content is neither created nor endorsed by Google. Report Abuse - Terms of Service - Privacy Policy

Google Forms
